# BCL-XL is an actionable target for treatment of malignant pleural mesothelioma

**DOI:** 10.1038/s41420-020-00348-1

**Published:** 2020-10-31

**Authors:** Surein Arulananda, Megan O’Brien, Marco Evangelista, Tiffany J. Harris, Nikita S. Steinohrt, Laura J. Jenkins, Marzena Walkiewicz, Robert J. J. O’Donoghue, Ashleigh R. Poh, Bibhusal Thapa, David S. Williams, Trishe Leong, John M. Mariadason, Xia Li, Jonathan Cebon, Erinna F. Lee, Thomas John, W. D. Fairlie

**Affiliations:** 1 Olivia Newton-John Cancer Research Institute, Heidelberg, VIC Australia; 2 School of Cancer Medicine, La Trobe University, Bundoora, VIC Australia; 3 Department of Medical Oncology, Austin Health, Heidelberg, VIC Australia; 4 Department of Clinical Pathology, University of Melbourne, Melbourne, VIC Australia; 5 Department of Pathology, Austin Health, Heidelberg, VIC Australia; 6 Department of Mathematics and Statistics, La Trobe University, Bundoora, VIC Australia; 7 Department of Biochemistry and Genetics, La Trobe Institute for Molecular Science, La Trobe University, Bundoora, VIC Australia; 8 Present Address: Department of Pharmacology and Therapeutics, University of Melbourne, Melbourne, VIC Australia; 9 Present Address: Peter MacCallum Cancer Centre, Melbourne, VIC Australia

**Keywords:** Targeted therapies, Apoptosis

## Abstract

Despite having one of the lowest survival rates of all cancers, there have been no new approved treatments for malignant pleural mesothelioma (MPM) in over a decade. Standard-of-care treatment relies on Cisplatin plus Pemetrexed chemotherapy. Here, we tested a suite of BH3-mimetic drugs targeting BCL-2 pro-survival proteins of the intrinsic apoptotic pathway. We found BCL-XL is the dominant pro-survival protein in a panel of cell lines in vitro, though potent, synergistic cell killing occurred with MCL-1 co-targeting. This correlates with high-level expression of BCL-XL and MCL-1 in cell lines and a large cohort of patient tumour samples. BCL-XL inhibition combined with Cisplatin also enhanced cell killing. In vivo BCL-XL inhibition was as effective as Cisplatin, and the combination enhanced tumour growth control and survival. Genetic ablation of MCL-1 also enhanced the effects of BCL-XL inhibitors, in vivo. Combined, these data provide a compelling rationale for the clinical investigation of BH3-mimetics targeting BCL-XL in MPM.

## Introduction

Malignant pleural mesothelioma (MPM) is an aggressive and incurable, asbestos-related cancer associated with a high global mortality rate^1^. Despite rapid advances across multiple other tumour types, the only progress made in MPM treatment since 2003 is a modest improvement in median overall survival (OS) (by 2 months) with the addition of Bevacizumab to Cisplatin and Pemetrexed chemotherapy following the phase III MAPS study^2^. Multiple attempts to target some commonly mutated pathways in MPM with novel agents have been unuccessful^3^, whilst immune checkpoint inhibitors have had variable results, and are associated with increased toxicities^4^.

Defective apoptotic signalling is one of the hallmarks of cancer, and is associated with chemo-resistance^5^. Accordingly, one avenue for cancer treatment gaining momentum is to directly target the intrinsic apoptosis pathway governed by the BCL-2 protein family^6^. This is now possible due to the development of small molecule inhibitors, known as BH3-mimetics, which directly target the BCL-2 pro-survival proteins. ABT-737, and its orally bioavailable analogue ABT-263/“Navitoclax”, were the first bona fide BH3-mimetics described. These bind to BCL-2, BCL-XL, and BCL-W with high affinity^7,8^. Pro-survival protein-specific inhibitors have since been developed including ABT-199 (“Venetoclax”) which targets BCL-2^9^, A-1331852, a BCL-XL-specific inhibitor^10^, and S63845 and AZD5991, both MCL-1 inhibitors^11,12^, amongst others^13^. Venetoclax was recently approved by various regulatory bodies for use in the treatment of refractory chronic lymphocytic leukaemia^14,15^, and is being trialled in a range of other haematological cancers^15–18^.

Notably, single agent efficacy of BH3-mimetics is predominantly observed across haematological malignancies as compared to solid cancers. Navitoclax showed promising activity in small-cell lung cancer mouse xenograft models, but when tested in a phase II human clinical trial, there was limited benefit seen and this was associated with dose-limiting toxicity (thrombocytopenia) due to potent, on-target killing of platelets^19^. In other solid cancers BCL-XL is frequently identified as a key pro-survival protein, although a common theme that is emerging is their additional co-dependence on MCL-1. This presents issues for translation to the clinic as in vivo co-administration of BCL-XL and MCL-1 inhibitors in mice results in fatal hepatotoxicity^20^.

Studies on the importance of the intrinsic apoptosis pathway in MPM are relatively limited. Expression analyses on small patient cohorts and cell lines have been contradictory, although expression of BCL-XL and MCL-1 is consistently reported. The investigation of BH3-mimetics in MPM has also been limited^21,22^, though a recent study demonstrated single-agent efficacy with the BCL-XL inhibitor, A-1331852, which was enhanced by co-treatment with ionising radiation^23^.

In this current study, we show MPM cells are predominantly dependent on BCL-XL but MCL-1 is also a barrier to drug responses and, for the first time, in vivo responses can be achieved in MPM cell xenografts with BCL-XL inhibitors. Hence, BH3-mimetics have potential for future clincial application in the treatment of MPM.

## Results

### BCL-2 family protein expression in MPM cell lines

**Fig. 1:**
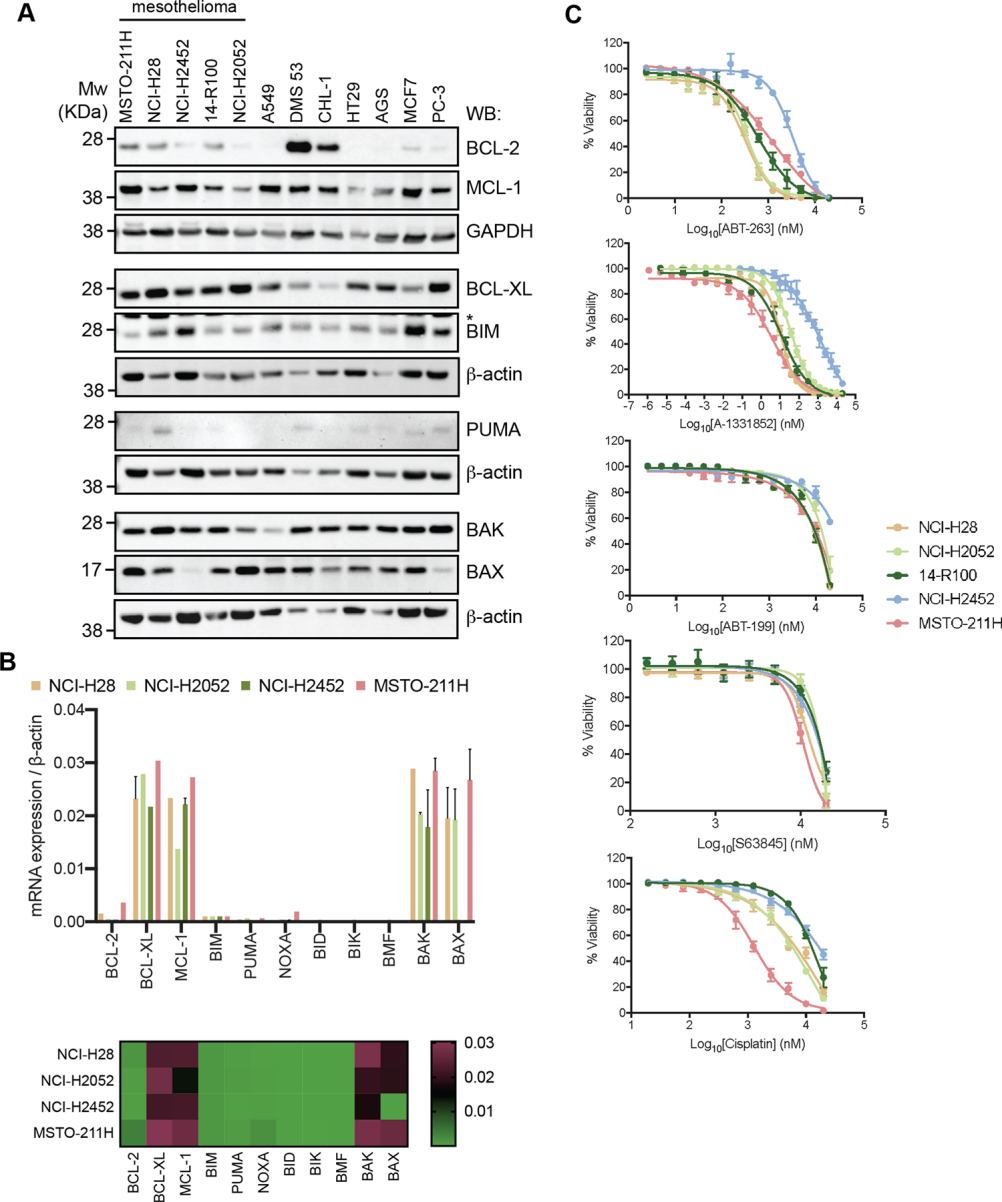
Expression of BCL-2 family members in MPM cell lines and their sensitivity to BH3-mimetics. **A** Cell lysates from established, and patient-derived MPM and other solid tumour cell lines were analysed by Western blot and probed for pro-survival or pro-apoptotic BAK/BAX and BH3-only proteins. Blots were re-probed for GAPDH or β-actin as a loading control. * Indicates non-specific band. **B** Relative mRNA levels for a wider set of BCL-2 genes were determined for a subset of cell lines by qRT-PCR, and generally reflect the protein expression pattern. Heatmap summarises this data. **C** Single agent activity of BH3-mimetics in MPM cell lines as determined by CellTiter-Glo viability assays after 72 h treatment. Drugs targeting BCL-XL were most effective. Data represent mean ± SEM (*n* = 3).

BCL-2 family member expression was compared in cell lines representative of the major MPM subtypes to their expression in other solid tumour cell lines by Western blot. Of the pro-survival proteins, BCL-XL was strongly expressed in all the MPM cell lines at levels relatively higher than most other tumour cell lines, whilst MCL-1 levels were consistent with the others (Fig. 1A). BCL-2 was weakly expressed in all MPM cell lines and all other lines examined except DMS53 (small-cell lung cancer) and CHL-1 (melanoma). Pro-apoptotic effector proteins BAK and BAX were readily detected in most cell lines, as was the BH3-only protein BIM, whilst PUMA expression was variable. These data were consistent with mRNA levels assessed by qRT-PCR in a subset of MPM cell lines probing for a wider panel of BCL-2 family members (Fig. 1B).

### Targeting BCL-XL effectively reduces MPM cell line viability in vitro

Human MPM cell lines were treated with ABT-263 (targeting BCL-2, BCL-XL, BCL-W), ABT-199 (targeting BCL-2), A-1331852 (targeting BCL-XL) and S63845 (targeting MCL-1), and cell viability determined using CellTiter-Glo assays that reflect total live cell numbers. All cell lines were relatively resistant to both ABT-199 and S63845 (EC_50_ values > 10 μM) (Fig. 1C; see Table S2 for all EC_50_ values). However, potent cell killing of most cell lines was observed with A-1331852 (EC_50_ values < 50 nM), and to a lesser extent ABT-263 (EC_50_ < 1 μM). All cell lines were also were relatively resistant to the standard-of-care drug Cisplatin (EC_50_ > 5 μM) except MSTO-211H (EC_50_ ~ 1.5 μM). Hence, the sensitivity of the cell lines to A-1331852 and ABT-263, suggests BCL-XL is the more critical pro-survival protein in MPM.

### Co-targeting of BCL-XL and MCL-1 synergistically reduces MPM cell viability in vitro

**Fig. 2:**
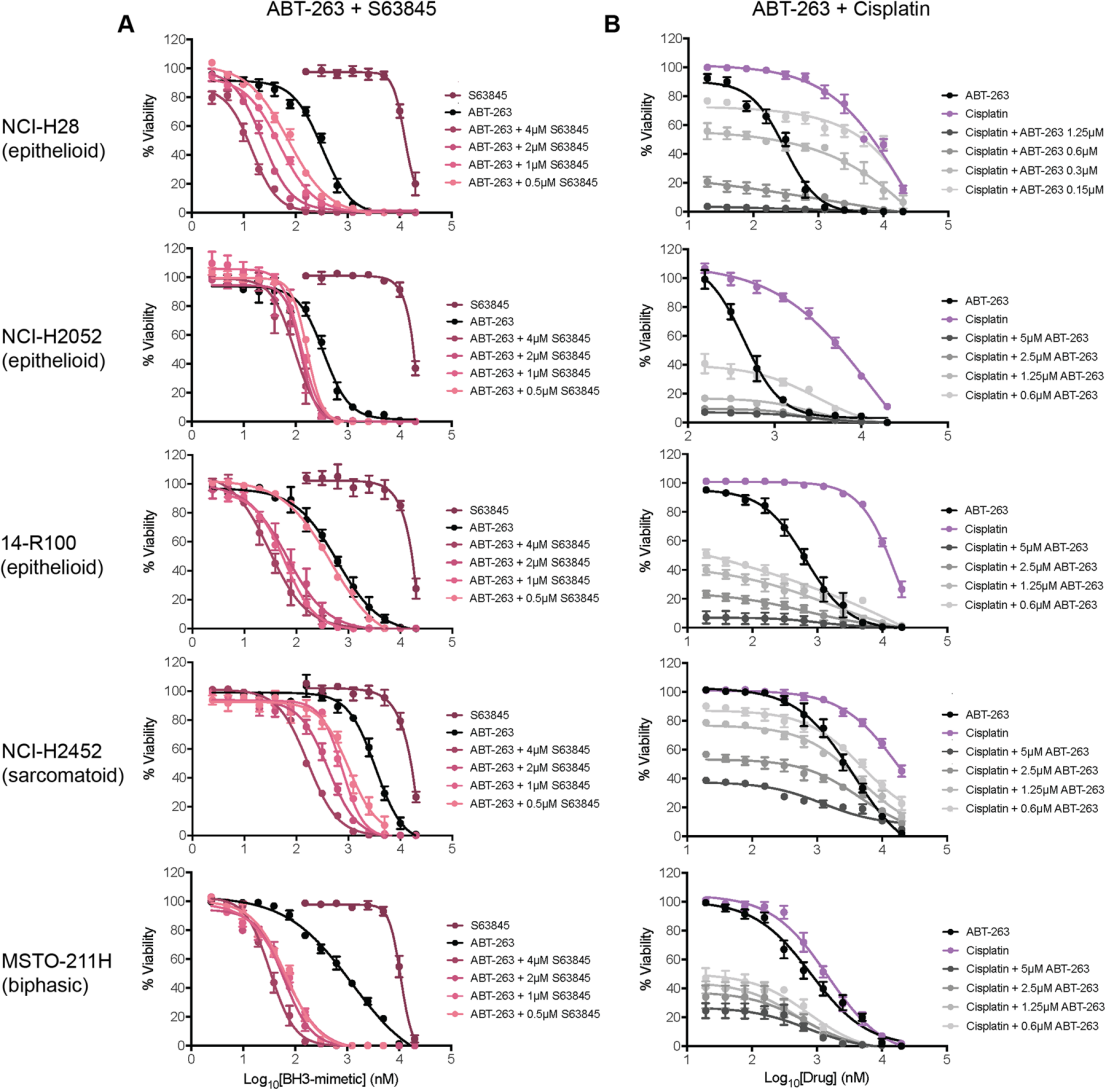
Drug combination studies with ABT-263. Co-treatment of MPM cells with ABT-263 and either **A** S63845 or **B** Cisplatin enhances responses over ABT-263 alone. Cell viability was determined using CellTiter-Glo assays after 72 h treatment. Data represent mean ± SEM (*n* = 3).

Although S63845 had only weak activity on MPM cell lines, MCL-1 is a well-established resistance factor for ABT-263 and A-1331852^24^. As MCL-1 was expressed in all MPM lines, we next tested BH3-mimetic combinations with S63845 to determine whether MCL-1 has any role in maintaining MPM cell survival, as in other solid cancers^24^. Indeed, potent cell killing (sub-nM EC_50_ values in some cases) was observed when ABT-263 or A-1331852 were titrated with S63845, with EC_50_ values decreasing by up to 100-fold (Figs. 2A, S1A and Table S2). These responses were shown to be synergistic (Fig. S2). In contrast, S63845 had minimal impact on ABT-199 activity (Fig. S3, Table S2), though some enhanced responses were observed and shown to be synergistic (Fig. S2). Combined, these data demonstrate that for maximal cell killing with BCL-XL inhibition in MPM, MCL-1 must also be antagonised.

### Combining Cisplatin with BCL-XL inhibition leads to increased cell killing in some MPM cell lines

Standard-of-care chemotherapy Cisplatin had relatively weak activity on most MPM cell lines (Fig. 1C), however, when combined with ABT-263 or A-1331852 its potency increased in most MPM lines (Figs. 2B and S1B, Table S3). Some synergy occurred between the drugs, though not to the same extent as with the S63845 (Figs. S2 and S4). Consistent with BCL-XL being the key pro-survival protein in MPM, ABT-199 (up to 5 μM) and S63845 (up to 4 μM) did not enhance cell killing by Cisplatin (Fig. S5A, B; Table S3). Hence, these data demonstrate that BCL-XL expression can blunt MPM responses to chemotherapy.

### Combination targeting of BCL-2 family proteins family induces apoptotic cell death

**Fig. 3:**
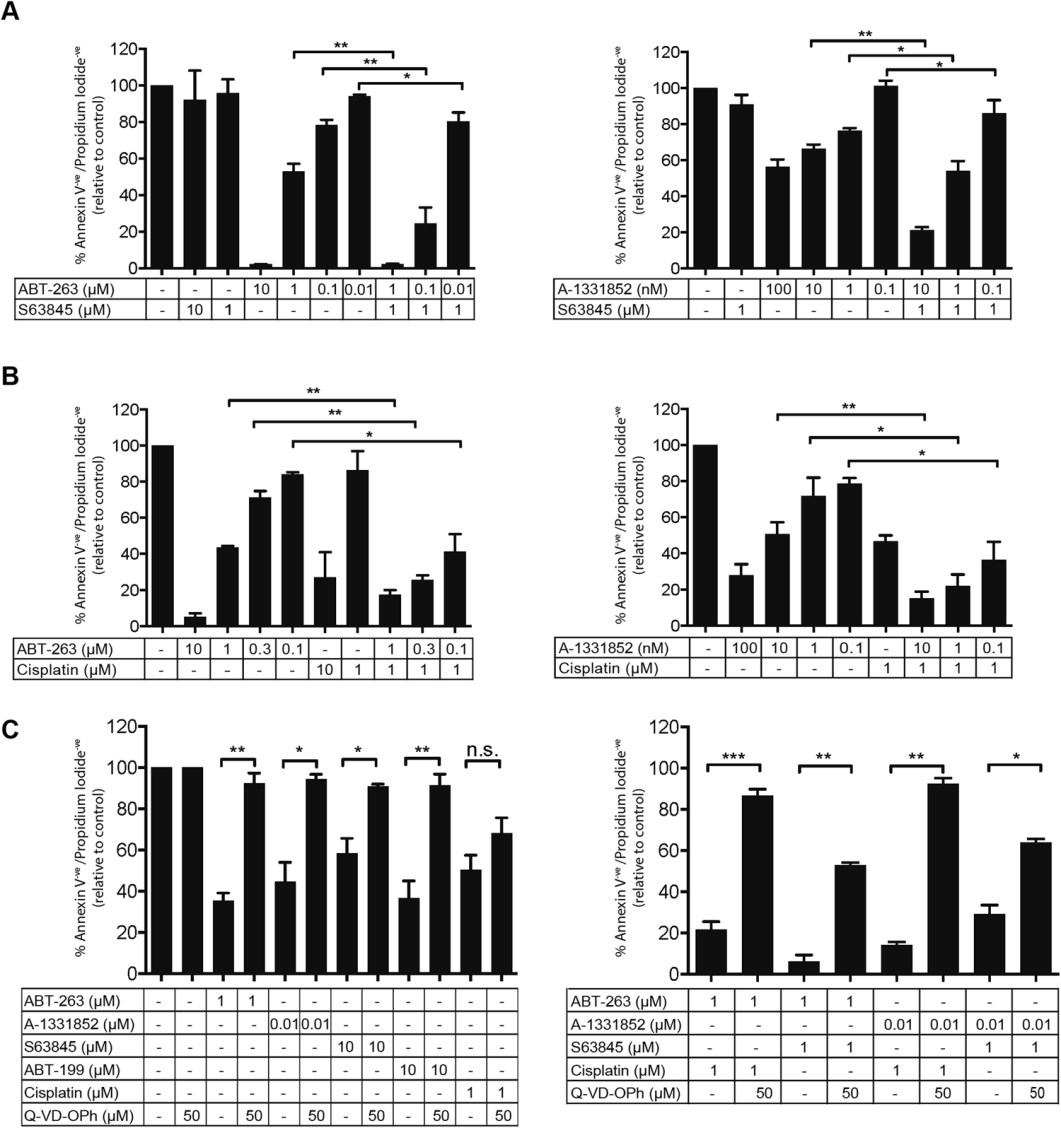
BH3-mimetic combinations reduce cell viability by inducing apoptosis. MSTO-211H cells were treated with ABT-263 or A-1331852 for 72 h in the presence or absence of **A** S63845 or **B** Cisplatin and apoptosis induction monitored by FACS using Annexin V/propidium iodide staining. **C** Apoptosis induction was confirmed for drugs as single agents (left panel) or in combination (right panel), by treating cells in the presence of the pan-caspase inhibitor Q-VD-OPh. Data represent mean ± SEM (*n* = 3) *p ≤ 0.05, **p ≤ 0.01 and ***p ≤ 0.001 (unpaired Students *t*-test).

CellTiter-Glo assays reflect overall live cell numbers, hence, are influenced by cell proliferation and death. To provide insight into apoptosis induction by BH3-mimetics, we performed FACS-based assays on two MPM lines using Annexin V/propidium iodide exclusion as a measure of cell viability. Significant loss of viability was observed across drug doses and combinations (Figs. 3A, B and S6A, B), with close correlation to the CellTiter-Glo assays. Addition of the pan-caspase inhibitor (Q-VD-OPh) significantly increased cell viability (Figs. 3C and S6C), consistent with apoptotic cell death in MPM cells by BH3-mimetics.

### BCL-XL inhibition in combination with Cisplatin in vivo leads to improved tumour growth control and significant improvement in survival

**Fig. 4:**
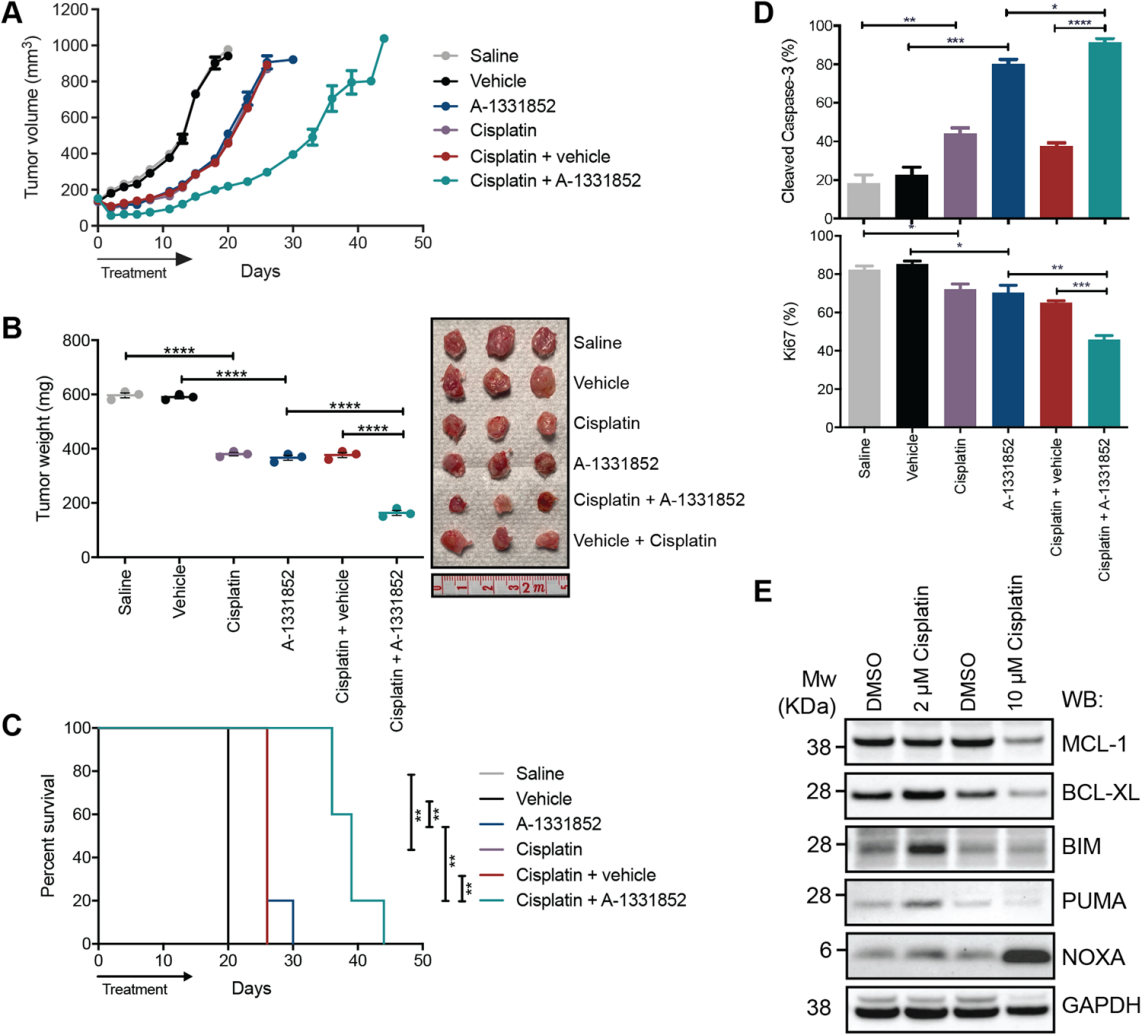
BCL-XL inhibition results in tumour growth control in MPM xenografts. **A** MSTO-211H xenograft tumour volumes measured during and following treatment with A-1331852 (25 mg/kg, 14 days by oral gavage) and Cisplatin (4 mg/kg, once on day 1 intraperitoneal injection), the combination, or appropriate controls. **B** Tumour masses at Day 19 (i.e. 1 day after the final dose of A-1331852 or vehicle administration). Each point is the mass of an individual tumour (photographed) with the bar indicating the mean ± SEM (*n* = 3) significance determined by Student’s *t*-test (unpaired). **C** Kaplan–Meier survival curves with log-rank analysis of mice treated with A-1331852, Cisplatin and combinations of both, with relevant vehicle controls. Survival endpoint was when tumours reached 1000 mm^3^ as dictated by the ethics approval associated with this experiment. Significance determined by Log-rank (Mantel–Cox test). **D** Immunohistochemistry analysis of tumours for cleaved Caspase-3 and Ki67. Values represent the mean % positively stained cells for each antibody in five different fields of view. Data are mean ± SEM (*n* = 3), significance determined by Student’s *t*-test (unpaired). **E** Responses of MSTO-211H cells to Cisplatin determined by Western blot. Cell lysates were prepared 72 h after treatment with indicated concentrations of Cisplatin and Western blots probed with antibodies to the indicated BCL-2 family proteins. Western blots were reprobed with antbody to GAPDH as a loading control. ****p ≤ 0.0001, ***p ≤ 0.001, **p ≤ 0.01, *p ≤ 0.05.

We next assessed whether the potent single-agent activity of A-1331852 could be recapitulated in vivo, and how it compared with standard-of-care treatment, Cisplatin. Cisplatin and A-1331852 resulted in similar tumour growth control and survival (i.e. time to ethical endpoint tumour size of 1000 mm^3^; Fig. 4A–C) relative to respective vehicle controls. Strikingly, the combination of BCL-XL inhibition and Cisplatin further extended disease control, with a median survival time (39 days) significantly longer compared to either drug alone (26 days, *p* < 0.01) (Fig. 4C), and almost double that of controls (20 days, *p* < 0.01).

Drug-treated tumours had increased apoptotic cell numbers (cleaved Caspase-3-positive), accompanied by reduced proliferating (Ki67-positive) cell numbers (Fig. 4D). Treatments were well tolerated, without obvious toxicity or weight loss (<15%) (Fig. S7A). Mice treated with A-1331852 had significantly reduced platelets when analysed 24 h after dosing, consistent with BCL-XL being important in platelet survivival, though normalised within a week of treatment cessation (Fig. S7B).

Immunohistochemistry and qRT-PCR showed BIM levels (protein and mRNA) increased in tumours from mice receiving Cisplatin, and unexpectedly, MCL-1 levels were reduced in tumours following A-1331852 treatment (Fig. S8A, B). As the tumours were analysed several weeks after Cisplatin administration, we also examined more immediate effects of Cisplatin treatment in MSTO-211H cells in vitro (Fig. 4E). Surprisingly, the effect was dose-dependent. At 2 μM, the predominant effect was on BIM expression (as in the tumours), as well as PUMA, whilst at 10 μM, NOXA was most strongly induced. Hence, these data provide further clues as to how Cisplatin affects MPM tumour growth and co-operates with A-1331852 in vivo. Overall, these data illustrate that BCL-XL inhibition significantly impacts MPM tumour growth in vivo and enhances the efficacy of Cisplatin.

### ABT-263 inhibits MPM tumour growth in vivo and this is enhanced by Cisplatin co-treatment

As A-1331852 has yet to be used in humans, we next tested ABT-263 (Navitoclax) in vivo as it potentially provides an avenue for future clinical studies. Using a similar dosing schedule (albeit with higher doses), ABT-263 mirrored A-1331852 in terms of efficacy alone and in combination with Cisplatin (Fig. S9A–C), weight maintenance (Fig. S9D), reversible thrombocytopaenia, (Fig. S9E), and ex vivo analyses of markers of apoptosis (Fig. S9F), proliferation (Fig. S9G), and changes in BCL-2 family protein expression (Fig. S10A, B). Hence, these data further demonstrate that BCL-XL inhibition can significantly impact MPM tumour growth.

### BCL-XL inhibition together with *MCL-1* deletion leads to significant reduction in MPM tumour growth and survival benefit

Although BCL-XL and MCL-1 inhibition resulted in the most potent cell killing in MPM cell lines, combination treatment with BCL-XL and MCL-1 inhibitors results in acute fatal hepatotoxicity in mice^20^. To circumvent this issue, we instead evaluated a genetic approach used previously^20^. MSTO-211H cells were engineered to express Cas9 and doxycycline-inducible sgRNAs targeting *MCL-1*, which were both effective (Fig. S11A). *MCL-1* deletion did not effect cell viability or sensitivity to ABT-199 (consistent with combination data with S63845 and ABT-199), but sensitised the cells to A-1331852 by 14-22-fold, via apoptosis induction (Fig. S11B–D).

**Fig. 5:**
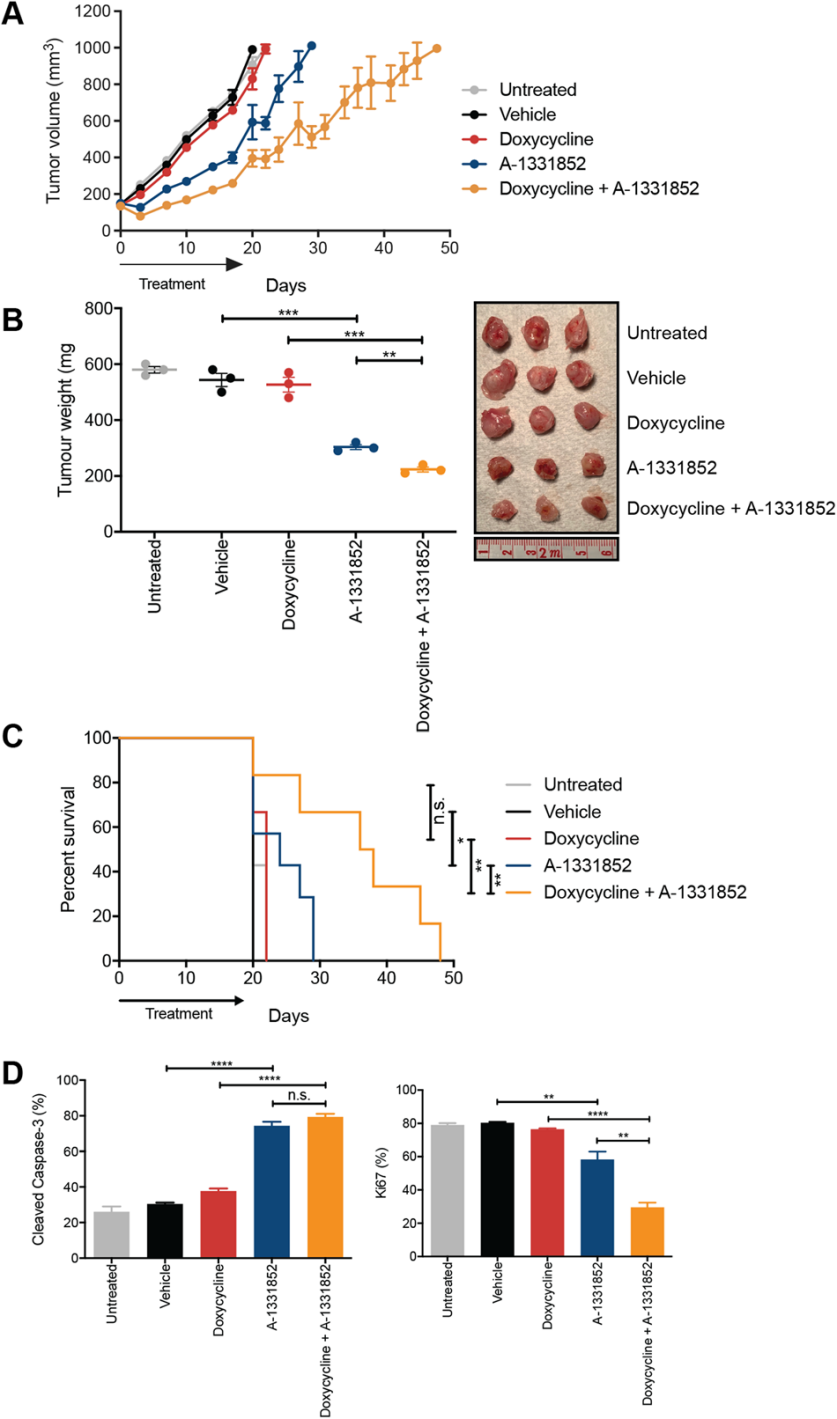
MCL-1 deletion increases responses to BCL-XL in vivo. **A** MSTO-211H cells (with Cas9/MCL-1 sgRNA) tumour volumes measured during and following treatment with A-1331852 (25 mg/kg, 14 days by oral gavage), feeding doxycycline-containing chow, or appropriate controls. **B** Tumour masses at Day 19 (i.e. 1 day after the final dose of A-1331852 or vehicle administration). Each point is the mass of an individual tumour (photographed) with the bar indicating the mean ± SEM (*n* = 3). Significance determined by Student’s *t*-test (unpaired). **C** Kaplan–Meier survival curves with log-rank analysis of mice treated with A-1331852, doxycycline and combinations of both, with relevant vehicle controls. Survival endpoint was when tumours reached 1000 mm^3^ as dictated by the ethics approval associated with this experiment. Significance determined by Log-rank (Mantel–Cox test). **D** Immunohistochemistry analysis of tumours for cleaved Caspase-3 and Ki67. Values represent the mean % postively stained cells for each antibody in five different fields of view. Data are mean ± SEM (*n* = 3), significance determined by Student’s *t*-test (unpaired). ****p ≤ 0.0001, ***p ≤ 0.001, **p ≤ 0.01, *p ≤ 0.05.

We next tested the cell line expressing sgRNA MCL-1-1 in vivo as it more effectively sensitised cells to BCL-XL inhibition. Deletion of *MCL-1* had no effect on tumour growth compared to controls, while A-1331852 treatment had the same outcome observed in previous experiments (Fig. 5A–C). Impressively, inhibition of BCL-XL combined with *MCL-1* deletion doubled survival time compared to controls. Ex vivo analysis showed treated tumours had increased apoptosis and decreased proliferation (Fig. 5D). No major changes in body weight or BCL-2 family member expression were observed following treatment, except confirmation of *MCL-1* deletion by doxycycline (Fig. S12). Hence, dual blockade of BCL-XL and MCL-1 could be an effective strategy for treating MPM.

### Correlation between BCL-2 family protein expression in patient MPM tumour samples and survival

**Fig. 6:**
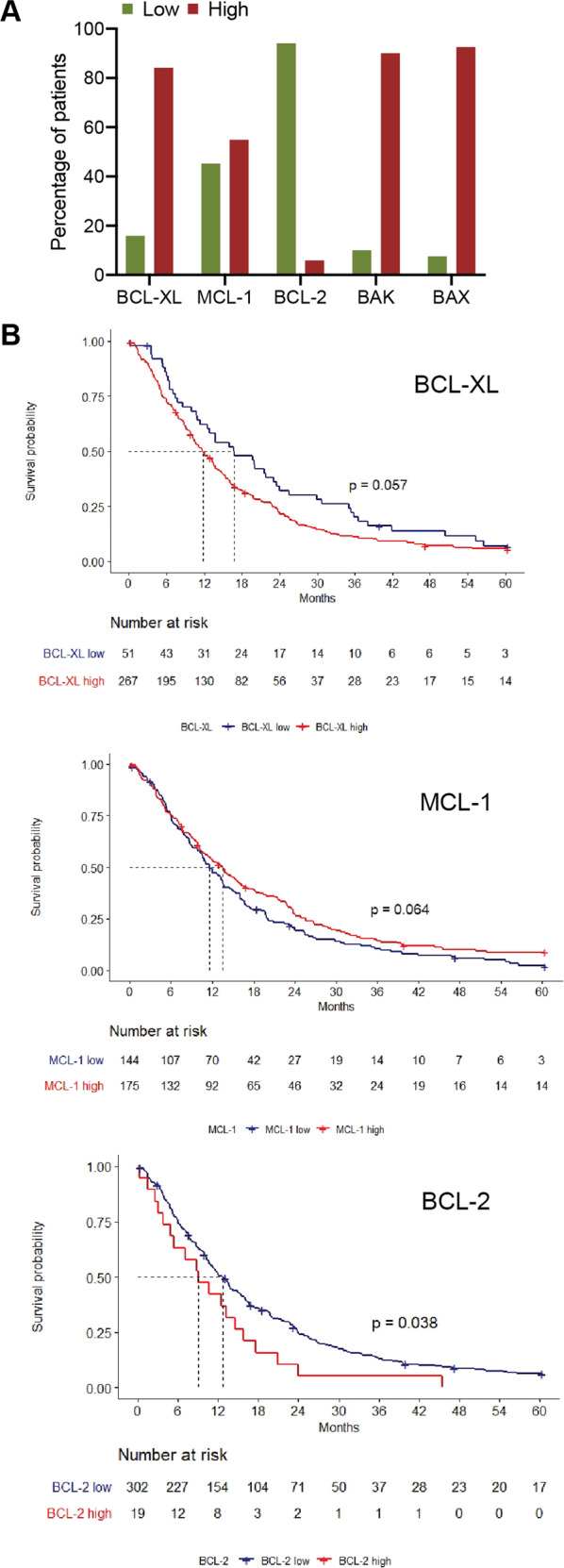
BCL-2 protein expression in MPM patient samples. **A** Quantitation of the percentage of patient tumour samples which have high or low expression of each BCL-2 family pro-survival protein (BCL-XL, MCL-1, and BCL-2) and pro-apoptotic protein (BAK and BAX) based on stratified H-scores. **B** Kaplan–Meier overall survival curves and correlation with expression of BCL-XL, MCL-1, BCL-2. Significance in Kaplan–Meier survival analysis was performed using the Mantel–Cox test.

As our studies to assess BH3-mimetic responses used MPM cell lines, it was important to confirm that their expression pattern of BCL-2 proteins was reflected in patient tumour samples. Accordingly, tissue microarrays representative of 326 individual patients with MPM (see ref. ^25^, and Table S4 for demographic and general patient data) were analysed for key pro-survival and pro-apoptotic family members. This patient dataset mirrored previous reports with a median OS of 12.5 months (Table S5) and disease stage (III and IV), Eastern Cooperative Oncology Group performance (status II–IV) and non-epithelioid histology being significantly associated with poorer outcomes (Fig. S13, Table S5)^25,26^, Immunohistochemical staining H-scores were subcategorised as high or low (based on the median score), and correlated with patient characteristics (Table S4). As with the Western blot analyses, high BCL-XL, MCL-1, BAK and BAX expression was observed in the majority (50–80%) of samples whilst a minority (~6%) of samples had BCL-2 expression (Fig. 6A). On univariate analysis, some general demographic and histological correlations were observed. For example, high BCL-XL expression was associated with age <65 years old (*p* = 0.016) but not sarcomatoid histology, which was also observed in another study with a much smaller sample size (*n* = 54)^27^. High MCL-1 expression was associated with non-sarcomatoid histology (*p* < 0.001), while high BCL-2 expression was associated with non-epithelioid histology (*p* < 0.001). For the pro-apoptotic proteins, BAK expression was associated with age <65 years (*p* = 0.018) and patients who had received surgery (*p* = 0.001). BAK and BAX expressions were both associated with non-sarcomatoid pathology (*p* = 0.004).

BCL-2 family protein expression was also correlated with patient OS (Fig. 6B, Table S6). Patients with highly expressing BCL-XL tumours had a non-significant, but strong trend towards inferior OS (11.8 versus 16.8 months) (HR 1.36, 95% CI 0.99–1.86, *p* = 0.057) but not high MCL-1 expression (13.5 versus 11.6 months) (HR 0.8, 95% CI 0.64–1.01, *p* = 0.064). Notably, patients with tumours with high BCL-2 expression had a significantly worse OS (9.1 versus 12.8 months) (HR 1.63, 95% CI 1.02–2.61, *p* = 0.038), though high BCL-2 was seen in only a small percentage of samples.

Finally, we analysed median OS for each pro-survival protein according to histology and found that the only statistically significant value was for high BCL-2 expression in patients with epithelioid MPM (8.76 versus 16.06 months) (HR 2.15, 95% CI 1.09–4.21, *p* = 0.023) (Fig. S14). When a multivariate Cox model was performed, histological subtype, advanced stage and receipt of systemic anti-cancer therapy remained statistically significant (Table S7).

These findings confirm that in a large cohort of MPM patients, BCL-2 expression (and potentially BCL-XL expression) are associated with poorer survival, regardless of histological subtype, the historically dominant prognostic factor in MPM.

## Discussion

Platinum-based chemotherapy, in combination with Pemetrexed, has been the mainstay of MPM treatment for close to two decades. Here, we provide the first compelling in vivo evidence that the intrinsic apoptotic pathway provides an actionable target worthy of consideration for future clinical investigation in MPM patients. Specifically, we demonstrated that BCL-XL is the critical BCL-2 pro-survival family member, though MCL-1 expression also provides a significant barrier to apoptotic induction using BH3-mimetic drugs.

The impressive MPM cell killing activity by BCL-XL inhibition using A-1331852 or ABT-263 we observed was consistent with recent in vitro studies using the same agents in MPM^23^. However, whilst there was no single-agent activity with S63845 (also recently shown)^23^, our studies are the first to demonstrate that MCL-1 and BCL-XL co-targeting with BH3-mimetics synergistically increases MPM cell killing in all MPM subtypes. These data agree with earlier work showing antisense oligonucleotides against BCL-XL induce apoptosis in MPM cells^28–30^, and studies showing RNAi against MCL-1 and BCL-XL co-operate to induce MPM cell killing, as does a NOXA peptide (targeting MCL-1) with ABT-737^21,31^. Our data are also consistent with the expression pattern of BCL-2 family proteins in our cell lines, as well as a large cohort of patient samples where BCL-XL and MCL-1 are the predominant pro-survival proteins expressed.

Notably, BCL-2 inhibition in vitro using Venetoclax was ineffective either alone or in combination with S63845. This is in keeping with the low expression of BCL-2 seen across our MPM cell lines and patient tissues as BCL-2 is a predictive biomarker for Venetoclax response^32,33^. Intriguingly, we found inferior OS in the high BCL-2 patient tumour samples, which unexpectedly was associated with the epithelioid subtype that generally has a better prognosis. Whilst, this represents a small proportion of patients (4.5%), there may be some benefit in future studies examining how higher BCL-2 expression influences responses to Venetoclax in MPM.

The single-agent activity of both A-1331852 and ABT-263 translated into the in vivo setting where both drugs were as effective as Cisplatin. A caveat here is that both BH3-mimetics were administered almost daily over the treatment period, whilst only a single dose of Cisplatin was used. Nevertherless, the BH3-mimetics were both well-tolerated (other than the expected on-target thrombocytopaenia), and indeed, pilot studies (not shown) using additional doses of Cisplatin had to cease due to excessive weight loss observed in the mice. Most promising was our data showing further improvement in tumour growth control and survival (essentially doubling) when both drugs were combined. This supports an earlier study that showed antisense oligonucleotides against BCL-XL promoted responses to Cisplatin in MPM xenografts^28^. Ex vivo analyses of tumours, and in vitro studies on the MSTO-211H cells used in xenografts, showed induction of BH3-only proteins BIM and NOXA, which would account for the improved outcomes seen in the combination studies, as both of these proteins can potently inhibit MCL-1. Unexpectedly, MCL-1 levels were also observed to be lower in tumours treated with BH3-mimetics, though this could be a consequence of apoptosis induction in the cells due to Caspase-3 activation, though it was not apparent in tumours treated with Cisplatin alone.

The improvement we observed with BCL-XL inhibitor *plus* Cisplatin combinations was reflected in our studies using tumour-specific *MCL-1* deletion to by-pass the fatal hepatotoxicity associated with co-administration of BCL-XL and MCL-1 inhibitors^20^. Although, this outcome is promising, the similarity in the outcomes of both of these “combination” in vivo experiments where indirect approaches were used to target MCL-1 (i.e. through BH3-only protein upregulation via chemotherapy or genetic deletion), suggests a subpopulation of cells within the tumours remain highly resistant to treatment as the tumours failed to regress. Whether this is due to inherent or acquired resistance is unclear at this stage, though will be an important avenue for future investigation. It will also be interesting to see whether these outcomes can be further improved once more targeted BH3-mimetics (e.g. through conjugation to tumour-specific antibodies) are developed and which could potentially allow safe co-treatment with BCL-XL *plus* MCL-1 direct inhibitors. One potential limitation of our mouse studies is that a single cell line was used, hence, future studies using additional cell lines, or alternatively, patient-derived xenografts, will be informative to provide further insight into the in vivo effect of BH3-mimics in MPM.

Currently, Navitoclax represents the most feasible BCL-XL inhibitor of those investigated for translation due to its previous (and current) testing in humans. One potential argument against this is the suboptimal responses and high rates of grade III–IV thrombocytopenia observed in the phase II small-cell lung cancer single arm study^19^. Platinum-relapsed small-cell lung cancer remains a notoriously difficult disease to treat and, therefore, this outcome should not be a deterrent towards testing Navitoclax in other cancers, such as MPM. Indeed, Navitoclax is currently being tested in several phase II clinical studies either as monotherapy or in novel combinations (i.e. non-small-cell lung cancer; NCT02079740, and melanoma; NCT01989585). Trials have also just commenced in both haematological and solid malignancies of a novel BH3-mimetic formulation targeting BCL-XL that may lead to reduced toxicity and increased efficacy (i.e. with AZD0466, NCT04214093). Hence, BCL-XL remains an actionable target for MPM treatment.

In conclusion, our data suggest that BCL-2 family proteins play a pivotal role in MPM cell survival. Although BCL-XL and MCL-1 co-inhibition provides the most effective strategy to eliminate MPM cells, BCL-XL inhibition alone has encouraging activity, and sensitises cells to Cisplatin chemotherapy, resulting in enhanced cell killing. In a disease with limited therapeutic options and poor survival outcomes, our findings will help direct relevant BH3-mimetic-based clinical trials for MPM patients.

## Materials and methods

### Drugs

All BH3-mimetic drugs and Cisplatin were purchased from Selleckchem.

### Immunohistochemical analysis

Tumour samples were collected from consensual patients with a diagnosis of MPM from the Austin Hospital (Victoria, Australia) between 1 January 2000 and 31 December 2010, under an Austin Hospital ethics committee-approved protocol (H2012/04446) and tissue microarray (TMA) prepared from 326 patient samples, as described previously^25^. Immunohistochemical analyses were performed as described previously^34^ using antibodies listed in Table S1A. Stained tissues were evaluated independently by two clinicians (S.A. and D.W. an accredited pathologist) and H-scores determined as described previously^25^.

### Cell culture

Cell lines were obtained from the ATTC (USA) except 14R-100, which was generated from a consensual Austin Hospital MPM patient (78-year-old male with epithelioid MPM, treatment-naïve) under Austin Health ethics number H2006/02394. All lines were confirmed to be mycoplasma negative based on in-house MycoAlert assays (Lonza). Cells were cultured in RPMI 1640 medium (Gibco) containing 10% (v/v) foetal calf serum (PAA Laboratories, Australia), 1% (v/v) GlutaMAX (Gibco) and 100 U/ml penicillin/100 mg/ml streptomycin (Gibco), and maintained at 37 °C with 5% CO_2_.

### Cell viability assays

CellTiter-Glo viability assays were performed after 72 h incubation with drugs as described previously^34^. For FACS-based assays, cells (30,000 per well) were seeded into 24-well plates, then 24 h later treated with vehicles, drugs, either alone, or in combination for 72 h. For pan-caspase inhibitor assays, Q-VD-OPh (MP Biomedicals) was added (final concentration 25 µM) to respective wells. Live and dead cells were harvested and pelleted by centrifugation then stained with Annexin V-APC (BD Biosciences) and propidium iodide (Sigma Aldrich) in Annexin V-binding buffer (BD Biosciences).

FACS analysis was performed on a BD FACSCanto II flow cytometer (BD Biosciences, USA). Data was analysed using FlowJo Software Version 10 (FlowJo-LLC, USA) with the number of viable cells (Annexin V negative/propidium iodide negative) normalised relative to the number of viable cells cultured in the vehicle control. GraphPad Software was used for statistical analysis. Synergy analysis was performed with Combenefit software using the BLISS models^35^.

### CRISPR/Cas-9 deletion of *MCL-1*

Deletion of MCL-1 was performed using the lentiviral vector system^36^, as described previously^34^. sgRNAs were designed using MIT CRISPR design software (http://crispr.mit.edu) providing the sequences: (1) 5′-CCGCCCGGGAGGGCGACTTT-3′ and (2) 5′-CTCAAAAGAAACGCGGTAAT-3′.

### Western Blot analysis

Cell lysates were prepared and analysed by Western blotting as described previously^34^. Membranes were probed with antibodies listed in Table S1B.

### Quantitative real-time polymerase chain reaction

Total RNA was extracted from cell pellets or fresh-frozen tumour samples and analysed by quantitative RT-PCR using previously published methods and primer sets^37^.

### Mouse xenograft experiments

All animal experiments were approved by the Austin Health Animal Ethics Committee (A2017_05465/A2018_05584) and carried out in accordance with the guidelines of Austin Health/University of Melbourne and conformed to the National Health and Medical Research Council’s code of practice for the care and use of animals for scientific purposes. Cells (4 × 10^6^) in Matrigel (BD Biosciences, Australia) were injected subcutaneously into the right flank of NOD-scid (6–8 weeks) female mice. When tumours reached 150–200 mm^3^, the mice were randomised to ensure consistent average tumour size across all study arms (*n* = 8–10 per group based on previous outcomes of drug treatment studies using MPM xenografts) and treated by oral gavage with either A-1331852 (25 mg/kg) or ABT-263 (100 mg/kg) prepared in 60% (v/v) Phosal 50PG, 27.5% (v/v) PEG400, 10% (v/v) ethanol, and 2.5% (v/v) DMSO for 5 consecutive days per week for 3 weeks. Cisplatin (4 mg/kg in phosphate-buffered saline) was delivered by single intraperitoneal injection on Day 1. To induce *Mcl-1* sgRNA expression, mice were fed with doxycyline chow (600 mg/kg; Specialty feeds, Australia). Tumours were measured using calipers by a blinded investigator (SA) and three mice per treatment group were culled by CO_2_ asphyxiation, 1 day after the final dose. The remaining mice were culled at ethical endpoint when tumours reached 1000 mm^3^. Blood was collected 24 h after treatment on day 10 and analysed on an Advia 2120 (Siemens).

### Statistical analyses

For patient characteristic data, OS was calculated from the time of initial diagnosis to death or last follow-up. Descriptive statistics were used for clinicopathological data. Comparisons of different parameters between the groups and BCL-XL, MCL-1, BCL-2, BAK, and BAX expression were performed using the Kruskal–Wallis one-way analysis of variance test for continuous variables, and Fisher’s exact test for categorical variables.

For both human survival analyses, univariate analysis were based on Kaplan–Meier analysis with the log-rank test for all the categorical variables, and then the multivariate Cox proportional hazard model was used. A *p*-value ≤ 0.05 was considered to indicate a statistically significant difference. Statistical analyses were performed by using the R statistical software packages.

For in vitro studies, data represent mean ± SEM (standard error of the mean). Student’s *t* tests were performed using GraphPad Prism software and applied to each experiment as described in the figure legends. For in vivo studies, significance in Kaplan–Meier survival analysis was performed using the Mantel–Cox test. A *p*-value ≤ 0.05 was considered significant, with *p < 0.05, **p < 0.01 and ***p < 0.001.

## Supplementary information

Supplementary Table Legends

Supplementary Table 1

Supplementary Table 2

Supplementary Table 3

Supplementary Table 4

Supplementary Table 5

Supplementary Table 6

Supplementary Table 7

Supplementary Figure Legends

Supplementary Figure 1

Supplementary Figure 2

Supplementary Figure 3

Supplementary Figure 4

Supplementary Figure 5

Supplementary Figure 6

Supplementary Figure 7

Supplementary Figure 8

Supplementary Figure 9

Supplementary Figure 10

Supplementary Figure 11

Supplementary Figure 12

Supplementary Figure 13

Supplementary Figure 14
