# Correction: BCL-XL is an actionable target for treatment of malignant pleural mesothelioma

**DOI:** 10.1038/s41420-020-00388-7

**Published:** 2021-06-15

**Authors:** Surein Arulananda, Megan O’Brien, Marco Evangelista, Tiffany J. Harris, Nikita S. Steinohrt, Laura J. Jenkins, Marzena Walkiewicz, Robert J. J. O’Donoghue, Ashleigh R. Poh, Bibhusal Thapa, David S. Williams, Trishe Leong, John M. Mariadason, Xia Li, Jonathan Cebon, Erinna F. Lee, Thomas John, W. Douglas Fairlie

**Affiliations:** 1 Olivia Newton-John Cancer Research Institute, Heidelberg, VIC Australia; 2 School of Cancer Medicine, La Trobe University, Bundoora, VIC Australia; 3 Department of Medical Oncology, Austin Health, Heidelberg, VIC Australia; 4 Department of Clinical Pathology, University of Melbourne, Melbourne, VIC Australia; 5 Department of Pathology, Austin Health, Heidelberg, VIC Australia; 6 Department of Mathematics and Statistics, La Trobe University, Bundoora, VIC Australia; 7 Department of Biochemistry and Genetics, La Trobe Institute for Molecular Science, La Trobe University, Bundoora, VIC Australia; 8 Present Address: Department of Pharmacology and Therapeutics, University of Melbourne, Melbourne, VIC Australia; 9 Present Address: Peter MacCallum Cancer Centre, Melbourne, VIC Australia

Correction to: *Cell Death Discovery*

10.1038/s41420-020-00348-1 published online 31 October 2020

The original version of this article unfortunately contained an error in the name of one of the authors (WD Fairlie). This has been corrected in the PDF and HTML versions. The original article has been corrected.

