# Supplementary material for: BCL-XL is an actionable target for treatment of malignant pleural mesothelioma
Source: Cell Death Discov. 2020 Oct 31;6:114. doi: 10.1038/s41420-020-00348-1 (PMC7603509; doi:10.1038/s41420-020-00348-1)
Supplement: Supplementary file 1 — Supplementary Table Legends [file 41420_2020_348_MOESM1_ESM.docx]

**SUPPLEMENTARY MATERIALS**

**Supplementary Tables**

**Table S1** Details of antibodies used in **A** Immunohistochemistry and **B** Western blotting

**Table S2** Summary of the EC_50_ values (in nM) obtained from BH3-mimetic drugs alone and in combination. Values were determined from CellTiter-Glo viability assays and represent mean ± SEM (n=3).

**Table S3** Summary of the EC_50_ values (in nM) obtained from BH3-mimetic drugs in combination with Cisplatin. Values were determined from CellTiter-Glo viability assays and represent the mean of n=3 separate assays.

**Table S4** Summary of BCL-2 family protein expression and individual patient characteristics.

**Table S5** Correlation between patient characteristics and median overall survival.

**Table S6** Correlation between BCL-2 family proteins expression and median overall survival.

**Table S7** Multivariate analysis for overall survival.
