# Supplementary material for: BCL-XL is an actionable target for treatment of malignant pleural mesothelioma
Source: Cell Death Discov. 2020 Oct 31;6:114. doi: 10.1038/s41420-020-00348-1 (PMC7603509; doi:10.1038/s41420-020-00348-1)
Supplement: Supplementary file 2 — Supplementary Table 1 [file 41420_2020_348_MOESM2_ESM.docx]

Table S1A: Antibodies used for immunohistochemistry

Table S1B: Antibodies used for Western blotting

| Antigen | Clone | Supplier / Catalogue Number |
| --- | --- | --- |
| BCL-XL | 54H6 | Cell Signalling Technology, cat# 2764 |
| MCL-1 | D5V5L | Cell Signalling Technology, cat# 39224 |
| BCL-2 | 124 | Cell Signalling Technology, cat# 15071 |
| BAK | D4E4 | Cell Signalling Technology, cat# 12105 |
| BAX | D2E11 | Cell Signalling Technology, cat#5023 |
| BIM | C34C5 | Cell Signalling Technology, cat# 2933 |
| Cleaved Caspase-3 | Asp175 | Cell Signalling Technology, cat# 9661 |
| Ki67 | 30-9 | Roche, cat# 790-4286 |

| Antigen | Clone | Supplier |
| --- | --- | --- |
| BCL-XL | 44 | BD Biosciences, cat# 610746 |
| MCL-1 | 19C4-15 | Walter and Eliza Hall Institute |
| BCL-2 | 7 | BD Biosciences, cat# 610539 |
| BAK | G317-2 | BD Biosciences, cat# 556382 |
| BAX | 6A7 | BD Biosciences, cat# 556467 |
| BIM | 3C5 | Walter and Eliza Hall Institute |
| PUMA | EP512Y | Abcam, cat# ab33906 |
| NOXA | 144C307 | Abcam, cat# ab13654 |
| β-actin | AC-74 | Sigma-Aldrich, cat# A5316 |
| GAPDH | D4C6R | Cell Signalling Technology, cat# #97166 |
