# Supplementary material for: BCL-XL is an actionable target for treatment of malignant pleural mesothelioma
Source: Cell Death Discov. 2020 Oct 31;6:114. doi: 10.1038/s41420-020-00348-1 (PMC7603509; doi:10.1038/s41420-020-00348-1)
Supplement: Supplementary file 5 — Supplementary Table 4 [file 41420_2020_348_MOESM5_ESM.docx]

**Supplementary Table S4.** Details of BCL-2 family protein expression and individual patient characteristics

| **Variables** | **BCL-X_L_** | **p** | **MCL-1** | **p** | **BCL-2** | **p** | **BAK** | **p** | **BAX** | **p** |
| --- | --- | --- | --- | --- | --- | --- | --- | --- | --- | --- |
| **Age, years** |  |  |  |  |  |  |  |  |  |  |
| Age <65  Age ≥65 | 13.1 (3.4)  11.6 (4.9) | 0.016 | 8.9 (5.5)  8.0 (5.7) | 0.154 | 1.0 (2.5)  1.1 (2.4) | 0.759 | 13.1 (3.3)  11.7 (3.6) | 0.018 | 14.2 (2.5)  13.8 (3.3) | 0.413 |
| **Sex** |  |  |  |  |  |  |  |  |  |  |
| Male  Female | 12.3 (4.3)  11.5 (5.1) | 0.357 | 8.3 (5.6)  8.9 (5.5) | 0.422 | 1.1 (2.5)  0.9 (2.1) | 0.956 | 12.6 (3.5)  12.7 (3.6) | 0.744 | 14.0 (3.0)  14.1 (3.3) | 0.528 |
| **Disease stage** | | |  |  |  |  |  |  |  |  |
| I-II  III-IV | 11.9 (4.4)  12.5 (4.5) | 0.109 | 8.4 (5.5)  8.3 (5.8) | 0.938 | 0.9 (2.4)  1.2 (2.5) | 0.203 | 12.6 (3.4)  12.6 (3.6) | 0.813 | 14.0 (3.1)  14.0 (3.0) | 0.659 |
| **ECOG performance status** | | | | | | |  |  |  |  |
| 0-1  2-4 | 12.4 (4.3)  11.8 (4.7) | 0.299 | 8.4 (5.5)  8.3 (5.8) | 0.961 | 1.1 (2.6)  1.0 (2.2) | 0.579 | 12.3 (3.7)  12.9 (3.1) | 0.255 | 14.0 (2.8)  13.9 (3.4) | 0.434 |
| **Smoking status (pack years)** | | | | | | | | | |  |
| Never  <20  ≥20 | 12.4 (4.3)  12.2 (4.2)  12.0 (4.8) | 0.884 | 8.1 (5.5)  8.8 (5.8)  8.5 (5.6) | 0.542 | 1.0 (2.1)  1.0 (2.6)  1.2 (2.7) | 0.577 | 12.3 (3.5)  13.1 (3.3)  12.6 (3.6) | 0.199 | 14.1 (2.8)  13.6 (3.7)  14.1 (2.9) | 0.317 |
| **Histological subtype** | | | | | | | | | | |
| Epithelioid  Sarcomatoid  Biphasic | 12.4 (4.4)  11.4 (4.6)  12.4 (4.3) | 0.267 | 9.7 (5.1)  4.0 (5.3)  7.3 (5.7) | <0.001  (S:B 0.02  S:E <0.001) | 0.8 (2.4)  1.4 (1.7)  1.7 (2.8) | <0.001  (E:B <0.001  E:S 0.001) | 13.0 (3.2)  11.0 (4.2)  12.3 (3.7) | 0.004  (S:E 0.003) | 14.3 (2.7)  12.8 (4.2)  13.9 (3.0) | 0.004  (S:E 0.004) |
| **Surgery** |  |  |  |  |  |  |  |  |  |  |
| Biopsy-only  EPP and P/D | 12.1 (4.5)  12.3 (4.4) | 0.685 | 8.4 (5.8)  8.4 (5.5) | 0.884 | 1.1 (2.4)  1.1 (2.4) | 0.780 | 12.0 (3.6)  13.1 (3.3) | 0.001 | 14.1 (3.0)  13.9 (3.1) | 0.383 |
| **Anti-cancer Therapy** | | |  |  |  |  |  |  |  |  |
| Chemotherapy  Radiotherapy  Chemo + radio  No therapy | 12.6 (3.9)  11.6 (5.3)  12.6 (3.5)  12.1 (4.5) | 0.921 | 9.2 (5.0)  9.4 (5.4)  9.5 (5.9)  7.7 (5.8) | 0.104 | 1.1 (2.4)  0.6 (1.2)  0.9 (1.3)  1.2 (2.7) | 0.544 | 12.9 (2.8)  12.3 (4.2)  13.0 (3.5)  12.5 (3.7) | 0.961 | 14.5 (2.1)  14.4 (1.9)  13.8 (3.0)  13.7 (3.5) | 0.229 |
