# Supplementary material for: BCL-XL is an actionable target for treatment of malignant pleural mesothelioma
Source: Cell Death Discov. 2020 Oct 31;6:114. doi: 10.1038/s41420-020-00348-1 (PMC7603509; doi:10.1038/s41420-020-00348-1)
Supplement: Supplementary file 6 — Supplementary Table 5 [file 41420_2020_348_MOESM6_ESM.docx]

**Supplementary Table S5.** Correlation between patient characteristics and median overall survival

| **Clinical**  **parameter** | **Data** | **Median OS**  **(95% CI; months)** | **Log-rank**  **(p-value)** |
| --- | --- | --- | --- |
| **Age, years** |  |  |  |
| Median (range)  Age <65 – no. (%)  Age ≥65 – no. (%) | 67 (24-88)  129 (39.6%)  197 (60.4%) | 12.5(10.8-13.8)  14.1(12.0-16.7)  10.9(9.1-13.7) | 0.066 |
| **Sex, n (%)** |  |  |  |
| Male  Female | 271 (83.1%)  55 (16.9%) | 12.5(10.6-14.1)  12.1(9.9-20.1) | 0.157 |
| **Disease stage, n (%)** | | | |
| I-II  III-IV | 158(48.5%)  168(51.5%) | 15.5(13.5-19.7)  8.8(7.0-10.9) | <0.001 |
| **ECOG Performance, n (%)** | | | |
| 0-1  2-4 | 198(60.7%)  128(39.3%) | 13.8(12.6-16.8)  8.7(6.3-12.1) | <0.001 |
| **Smoking history, n(%)** | | | |
| Never  <20  ≥20 | 145(44.5%)  73(22.4%)  108(33.1%) | 13.8(11.6-16.8)  12.5(8.8-15.2)  10.6(7.7-13.8) | 0.781 |
| **NLR, n** |  |  |  |
| Mean(sd) | 5.1(4.1) | Median(IQR) | 3.9(3.2) |
| **Histology, n (%)** | | | |
| Epithelioid  Sarcomatoid  Biphasic | 203(62.3%)  43(13.2%)  72(22.1%) | 15.7(13.5,19.8)  5.1(3.7-7.0)  10.9(9.6-13.7) | <0.001 |
| **Surgery, n (%)** |  |  |  |
| Biopsy-only  EPP or P/D | 162(49.8%)  163(50.2%) | 9.8(8.1-12.8)  13.7(12.0-16.1) | 0.124 |
| **Anti-cancer therapy, n (%)** | | | |
| Chemotherapy alone  Radiotherapy alone  Chemotherapy + radiotherapy  No therapy | 83(60.6%)  40(29.2%)  14(10.2%)  296 (68.4%) | 18.0(16.1-23.6)  12.0 (9.9-23.0)  23.9(18.4-NA)  8.6(7.0-10.6) | <0.001 |
