# Supplementary material for: BCL-XL is an actionable target for treatment of malignant pleural mesothelioma
Source: Cell Death Discov. 2020 Oct 31;6:114. doi: 10.1038/s41420-020-00348-1 (PMC7603509; doi:10.1038/s41420-020-00348-1)
Supplement: Supplementary file 7 — Supplementary Table 6 [file 41420_2020_348_MOESM7_ESM.docx]

**Supplementary Table S6.** Correlation between BCL-2 pro-survival family protein expression and median overall survival

| **Protein expression** | **Number of patients, (%)** | **Median OS**  **(95% CI; months)** | **Log-rank**  **(p-value)** |
| --- | --- | --- | --- |
| **BCL-XL** |  |  |  |
| BCL-XL High  BCL-XL Low | 269 (84.1%)  51 (15.9%) | 11.8 (11.3, 13.6)  16.8 (10.3, 24.0) | 0.057 |
| **MCL-1** |  |  |  |
| MCL-1 High  MCL-1 Low | 176 (54.8%)  145 (45.2%) | 13.5 (10.6, 16.2)  11.6 (10.2, 13.7) | 0.064 |
| **BCL-2** |  |  |  |
| BCL-2 High  BCL-2 Low | 19 (5.9%)  304 (94.1%) | 9.1 (5.3, 17.6)  12.7 (10.9, 14.2) | 0.038 |
| **BAK** | | | |
| BAK High  BAK Low | 288 (90.0%)  32 (10%) | 12.8 (10.9, 14.5)  11.1 (7.0, 21.9) | 0.700 |
| **BAX** | | | |
| BAX High  BAX Low | 296 (92.5%)  24 (7.5%) | 12.0 (10.7, 14.5)  13.3 (5.8, 28.0) | 0.891 |
