# Supplementary material for: BCL-XL is an actionable target for treatment of malignant pleural mesothelioma
Source: Cell Death Discov. 2020 Oct 31;6:114. doi: 10.1038/s41420-020-00348-1 (PMC7603509; doi:10.1038/s41420-020-00348-1)
Supplement: Supplementary file 8 — Supplementary Table 7 [file 41420_2020_348_MOESM8_ESM.docx]

**Supplementary Table S7.** Multivariate analysis for overall survival

| **Variables** | **Hazard ratio** | **Lower 95%** | **Upper 95%** | **p-value** |
| --- | --- | --- | --- | --- |
| Gender (male *vs* female) | 1.33 | 0.95 | 1.85 | 0.093 |
| Histological subtype (Epithelioid *vs* non-epithelioid) | 0.58 | 0.45 | 0.75 | <0.001 |
| Stage (III and IV *vs* I and II) | 1.51 | 1.19 | 1.91 | 0.001 |
| Anti-cancer therapy (no therapy *vs* chemotherapy,  radiotherapy and chemotherapy + radiotherapy) | 1.70 | 1.33 | 2.17 | <0.001 |
