# Supplementary material for: BCL-XL is an actionable target for treatment of malignant pleural mesothelioma
Source: Cell Death Discov. 2020 Oct 31;6:114. doi: 10.1038/s41420-020-00348-1 (PMC7603509; doi:10.1038/s41420-020-00348-1)
Supplement: Supplementary file 9 — Supplementary Figure Legends [file 41420_2020_348_MOESM9_ESM.docx]

**SUPPLEMENTARY MATERIALS**

**Supplementary Figures**

**Figure S1** Combination studies with A-1331852. Co-treatment of MPM cells with A-1331852 and either **A** S63845 or **B** Cisplatin enhances responses over A-1331852 alone. Cell viability was determined using CellTiter-Glo viability assays 72 hours after treatment. Data represent mean ± SEM (n = 3).

**Figure S2** Combinations of BH3-mimetics act synergistically. Synergy analysis was performed for BH3-mimetic drug combinations using Combenefit software with the BLISS synergy model.

**Figure S3** Combination studies with ABT-199 and S63845. Cell viability was determined using CellTiter-Glo viability assays 72 hours after treatment. Data represent mean ± SEM (n = 3).

**Figure S4** Synergy analysis of BH3-mimetics with Cisplatin co-treatments. Analysis was performed for BH3-mimetic drug combinations using Combenefit software with the BLISS synergy model.

**Figure S5** Combination studies of Cisplatin with ABT-199 and S63845. MPM cell lines were co-treated with Cisplatin and either **A** ABT-199 or **B** S63845. Cell viability was determined using CellTiter-Glo viability assays 72 hours after treatment. Data represent mean ± SEM (n = 3).

**Figure S6** BH3-mimetic combinations reduce cell viability by inducing apoptosis. NCI-H28 cells were treated with ABT-263 or A-1331852 in the presence or absence of **A** S63845 or **B** Cisplatin and apoptosis induction monitored after 72 hours by FACS using Annexin V / propidium iodide staining. Apoptosis induction was confirmed for **B** drugs as single agents or **D** in combination, by treating cells in the presence of pan-caspase inhibitor Q-VD-OPh. Data represent mean ± SEM (n = 3) *p ≤ 0.05, ** p ≤ 0.01 and *** p ≤ 0.001 (unpaired Students *t*-test).

**Figure S7** Effect on A-1331852 on mouse body weight and platelets**. A** Body weights of mice was measured during and after the treatment period. Data represent mean ± SEM (n=6-10). **B** Platelet count determined 24 hours post A-1331852, Cisplatin or vehicle dosing on Day 10 since start of treatment phase in all treatment groups and in Cisplatin *plus* A-1331852 treated mice 3 weeks (3w) post start of treatment phase. Data is mean ± SEM (n=3 mice per group), significance determined by Student’s *t*-test (unpaired). **** p≤0.0001, *p≤0.05.

**Figure S8** Effect of A-1331852 and Cisplatin on BCL-2 family expression. **A** H-scores for indicated BCL-2 family members. Sections were scored for staining by each antibody in 5 different fields of view. Data are mean ± SEM (n=3 tumors per group), significance determined by Student’s *t*-test (unpaired). **p≤0.01, *p≤0.05. **B** Expression of mRNA for BCL-2 family members in tumors were harvested at Day 19 of the experiment (1 day after final dose administration of A-1331852 or vehicle) determined by qRT-PCR. Data are mean ± SEM (n=3 tumors per group).

**Figure S9** ABT-263 treatment inhibits tumor growth control in MPM xenografts and this is enhanced by co-treatment with Cisplatin. **A** MSTO-211H cell tumor volumes measured during and following treatment with ABT-263 (25 mg/kg, 14 days by oral gavage) and Cisplatin (4 mg/kg, once by intraperitoneal injection), the combination, or appropriate controls. **B** Tumor masses at Day 19 (i.e. 1 day after the final dose of A-1331852 or vehicle administration). Each point is the mass of an individual tumor (photographed) with the bar indicating the mean ± SEM (n=3) significance determined by Student’s *t*-test (unpaired). **C** Kaplan-Meier survival curves with log-rank analysis of mice treated with A-1331852, Cisplatin and combinations of both, with relevant vehicle controls. Survival endpoint was when tumors reached 1000mm^3^ as dictated by the ethics approval associated with this experiment. Significance determined by Log-rank (Mantel-Cox test). **D** Body weights of mice (n=6-10) was measured during and after the treatment period. Data represent mean ± SEM (n=6-10). **E** Platelet count determined 24 hours post A-1331852 or vehicle dosing on Day 10 since start of treatment phase in all treatment groups and in Cisplatin *plus* A-1331852 treated mice 3 weeks (3w) post start of treatment phase. Data is mean ± SEM (n=3 mice per group), significance determined by Student’s *t*-test (unpaired). **F** Immunohistochemistry analysis of tumors for cleaved Caspase 3 (CC3) and Ki67. Values represent the mean % postively stained cells for each antibody in five different fields of view. Data are mean ± SEM (n=3), significance determined by Student’s *t*-test (unpaired). **** p≤0.0001, ***p ≤0.001, **p≤0.01, *p≤0.05.

**Figure S10** Effect of ABT-263 and Cisplatin on BCL-2 family expression. **A** H-scores for indicated BCL-2 family members. Sections were scored for staining by each antibody in 5 different fields of view. Data are mean ± SEM (n=3 tumors per group), significance determined by Student’s *t*-test (unpaired). **p≤0.01, *p≤0.05. **B** Expression of mRNA for BCL-2 family members in tumors were harvested at Day 19 of the experiment (1 day after final dose administration of ABT-263 or vehicle) determined by qRT-PCR. Data are mean ± SEM (n=3 tumors per group).

**Figure S11** Deletion of MCL-1 enhances sensitivity to BCL-XL inhibition. **A** MCl-1 is effectively deleted in MSTO-211H cells expressing Cas9 following induction sgRNA for MCL-1 with doxycycline (Dox). **B** As expected, deletion of MCl-1 increases sensitivity to BCL-XL inhibition with ABT-263 and A-1331852. Cell viability was determined following indicated treatments using CellTiter-Glo assays after 72 hours treatment. Data represent mean ± SEM (standard error of the mean) from n = 3 separate assays. **C** Tabulated EC_50_ for *MCL-1*-deleted and control cells following treatment with indicated BH3-mimetics determined from curves in **B**. Values represent the mean ± SEM (n=3). **C** BH3-mimetics reduce cell viability of *MCL-1*-deleted cell by induction of apoptosis measured by FACS following Annexin V/propidum iodide staining after treatment for 72 hours. Data are mean ± SEM (n=3 tumors per group).

**Figure S12.** Effect of A-1331852 treatment on BCL-2 family protein expression in *MCL-1*-deleted cells. H-scores for indicted BCL-2 family members. Sections were scored for staining by each antibody in 5 different fields of view. Data are mean ± SEM (n=3 tumors per group), significance determined by Student’s *t*-test (unpaired).

**Figure S13.** Kaplan-Meier overall survival curve and correlation with MPM histological subtype.

**Figure S14** Kaplan-Meier overall survival curves according to BCL-2 pro-survival proteins specified by histology. **A** BCL-XL **B** MCL-1 and **C** BCL-2 expression in epithelioid subtype and **D** BCL-XL **E** MCL-1 and **F** BCL-2 expression in non-epithelioid subtype.
