# Supplementary figures and images for: BCL-XL is an actionable target for treatment of malignant pleural mesothelioma
Source: Cell Death Discov. 2020 Oct 31;6:114. doi: 10.1038/s41420-020-00348-1 (PMC7603509; doi:10.1038/s41420-020-00348-1)

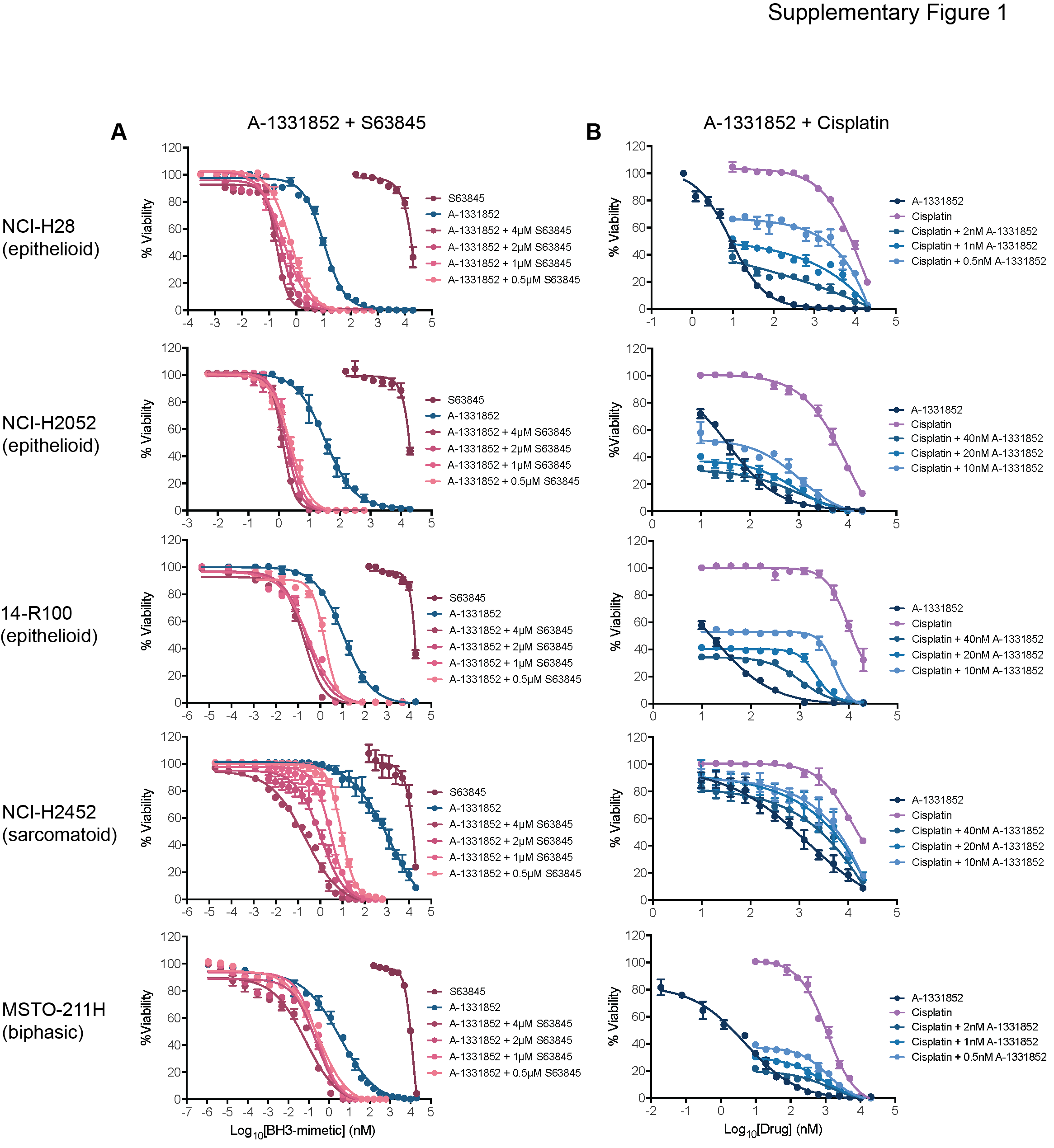

Supplement: Supplementary file 10 — Supplementary Figure 1 [file 41420_2020_348_MOESM10_ESM.png]

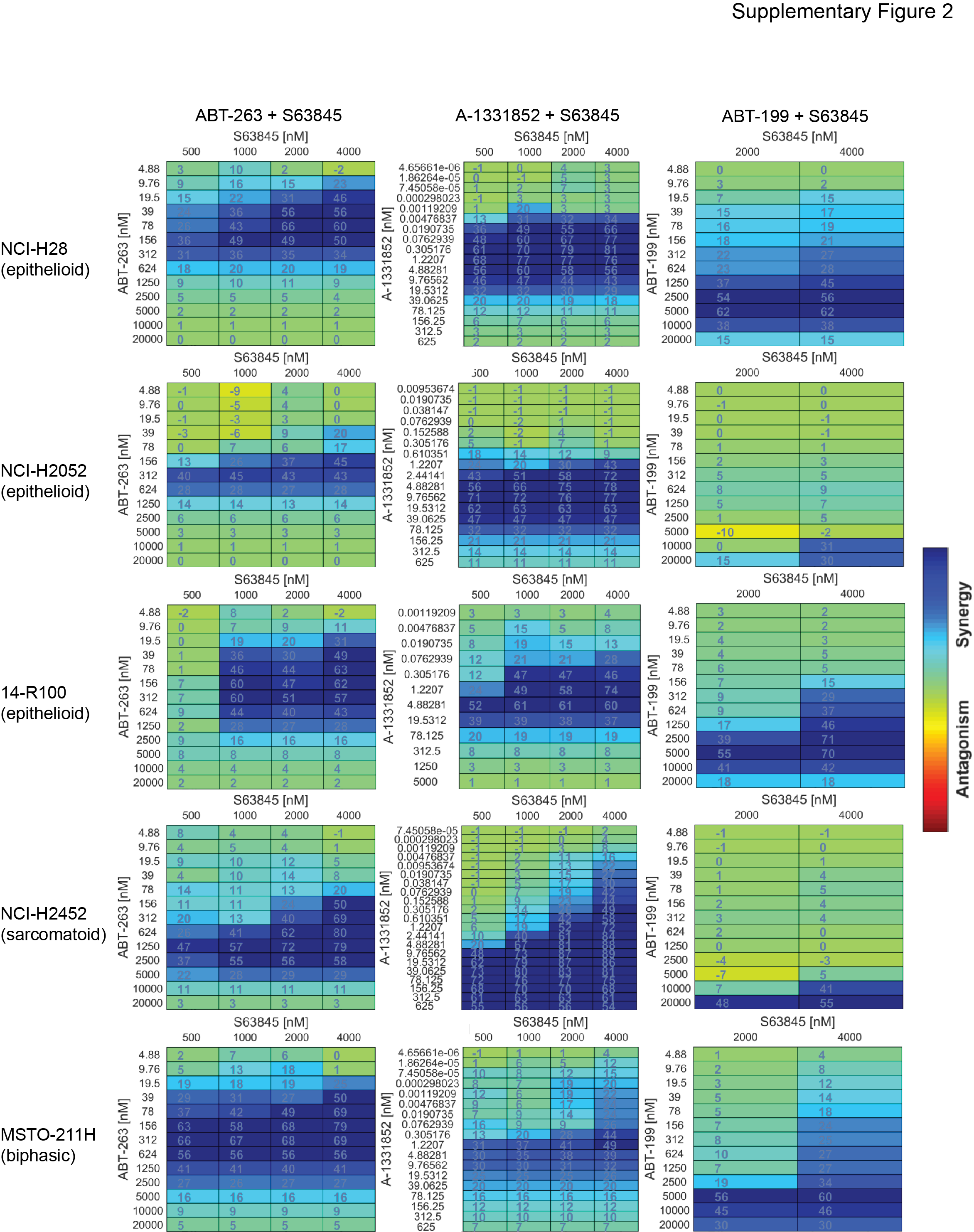

Supplement: Supplementary file 11 — Supplementary Figure 2 [file 41420_2020_348_MOESM11_ESM.png]

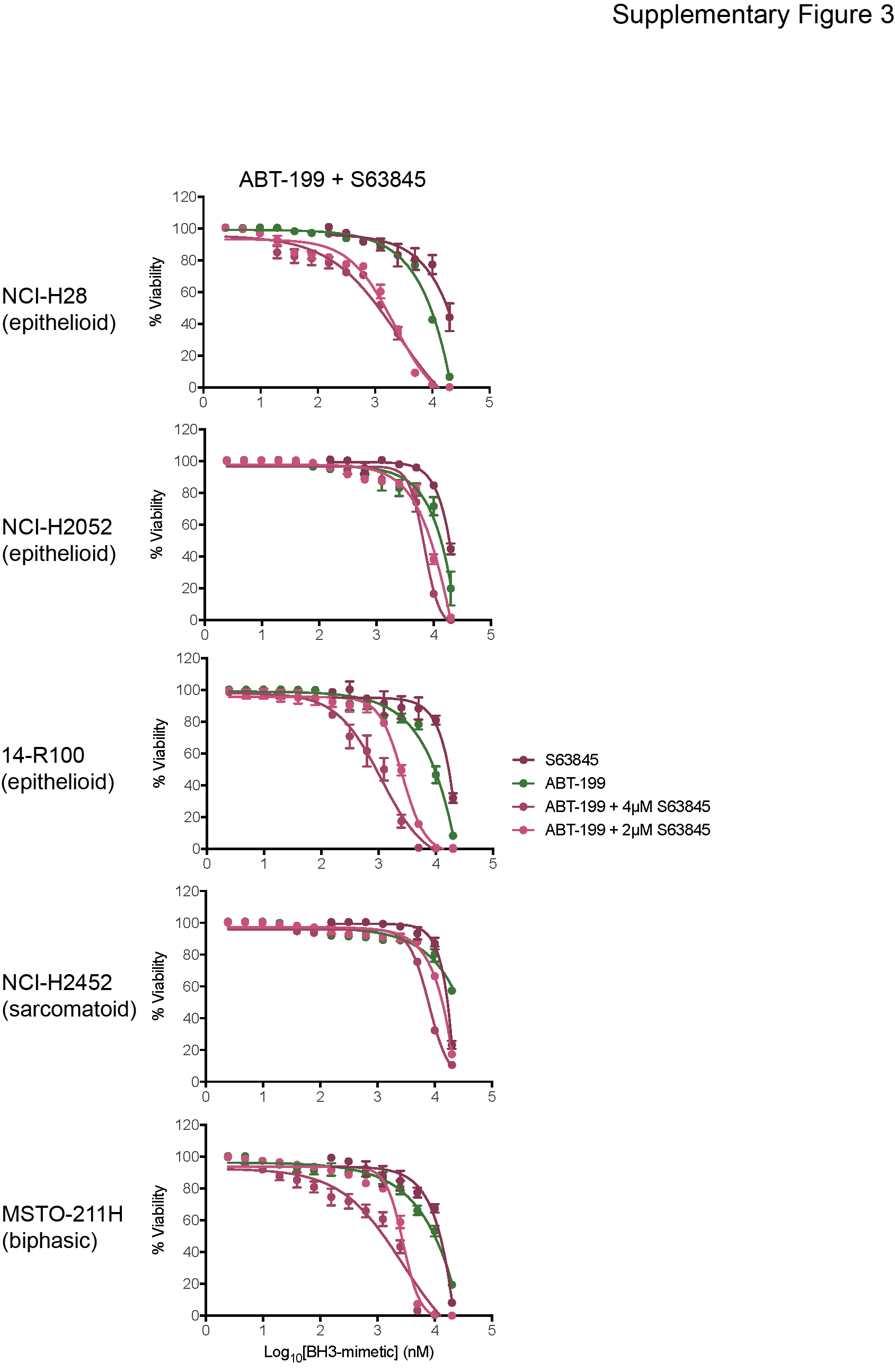

Supplement: Supplementary file 12 — Supplementary Figure 3 [file 41420_2020_348_MOESM12_ESM.png]

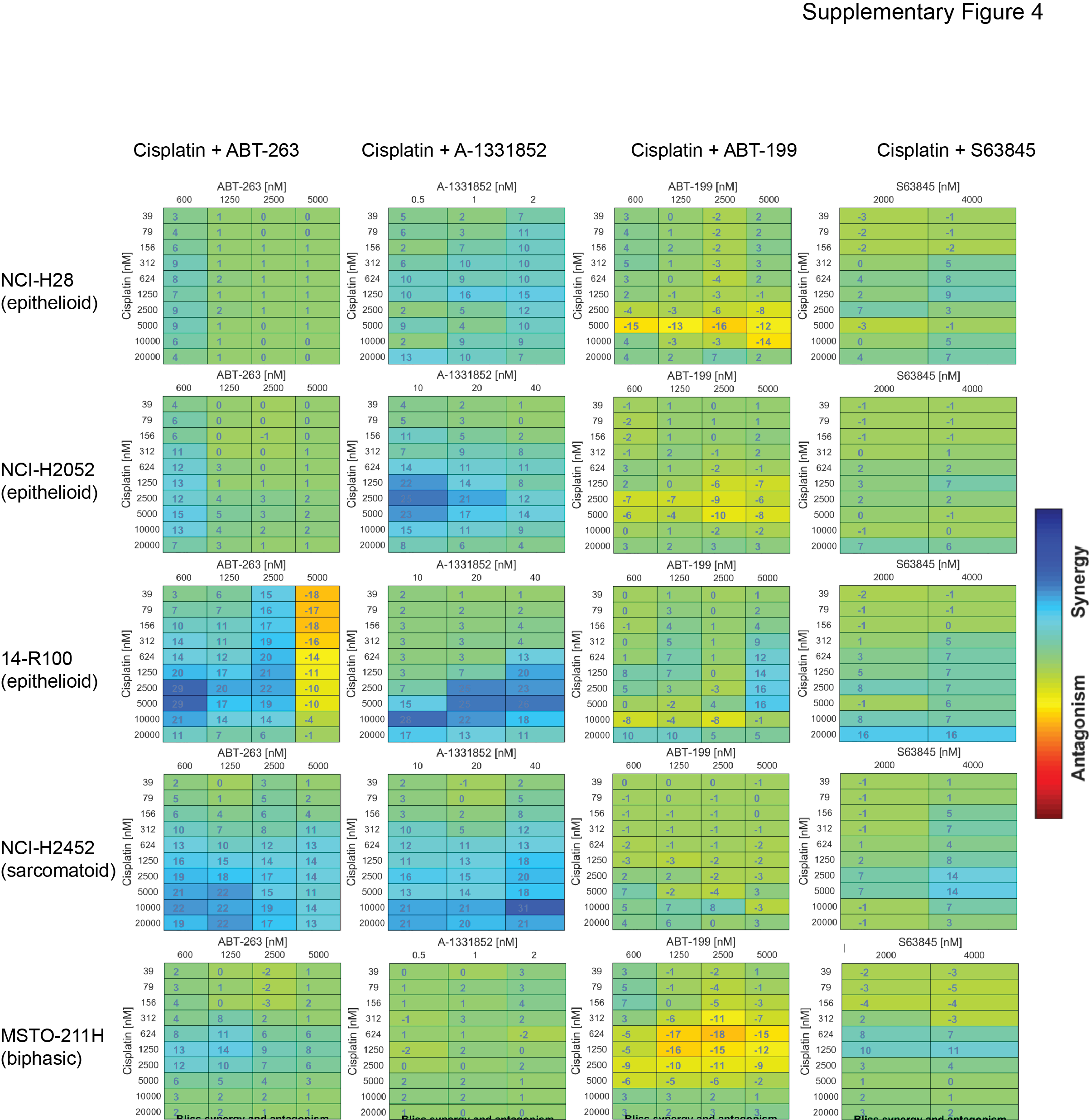

Supplement: Supplementary file 13 — Supplementary Figure 4 [file 41420_2020_348_MOESM13_ESM.png]

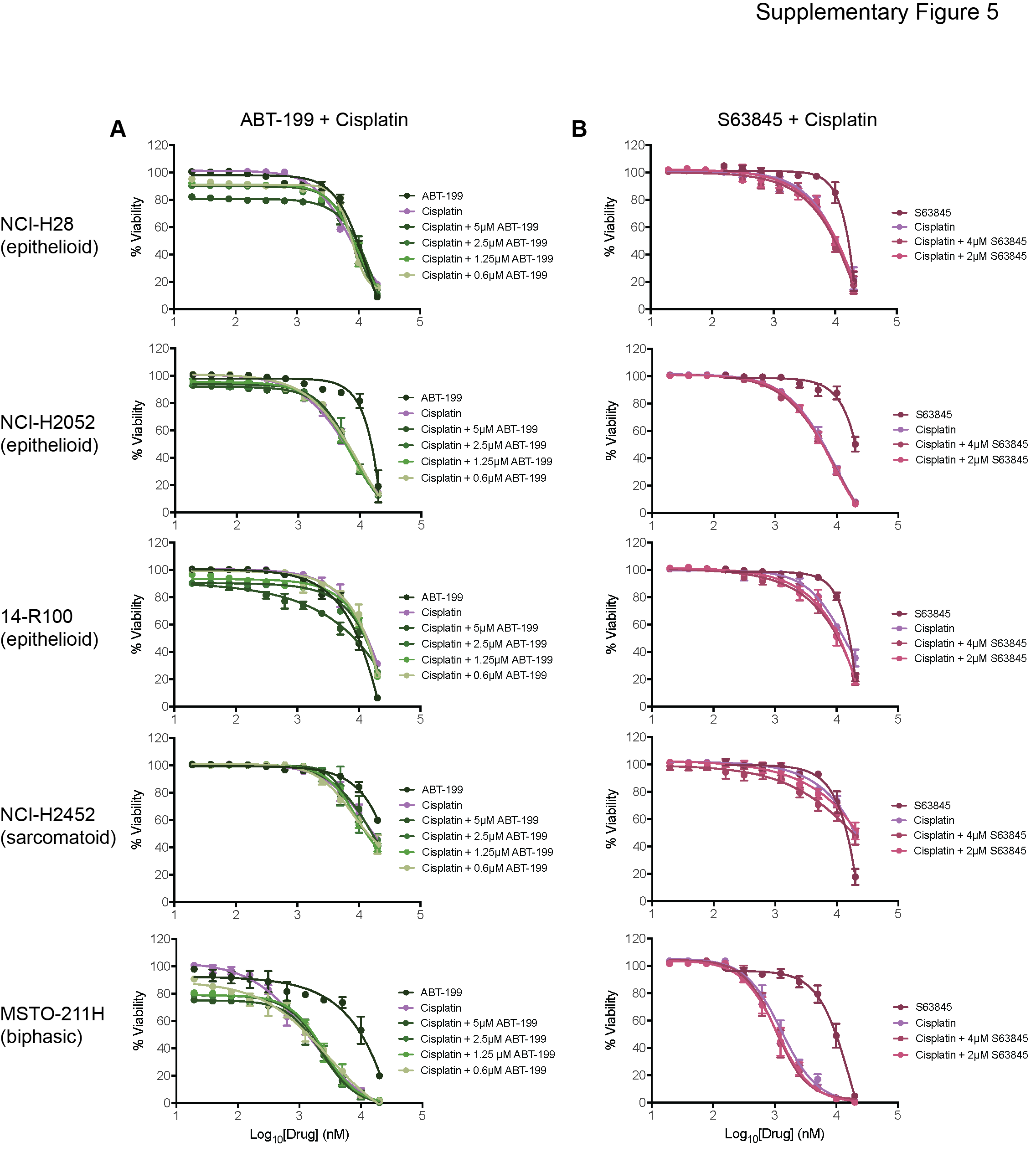

Supplement: Supplementary file 14 — Supplementary Figure 5 [file 41420_2020_348_MOESM14_ESM.png]

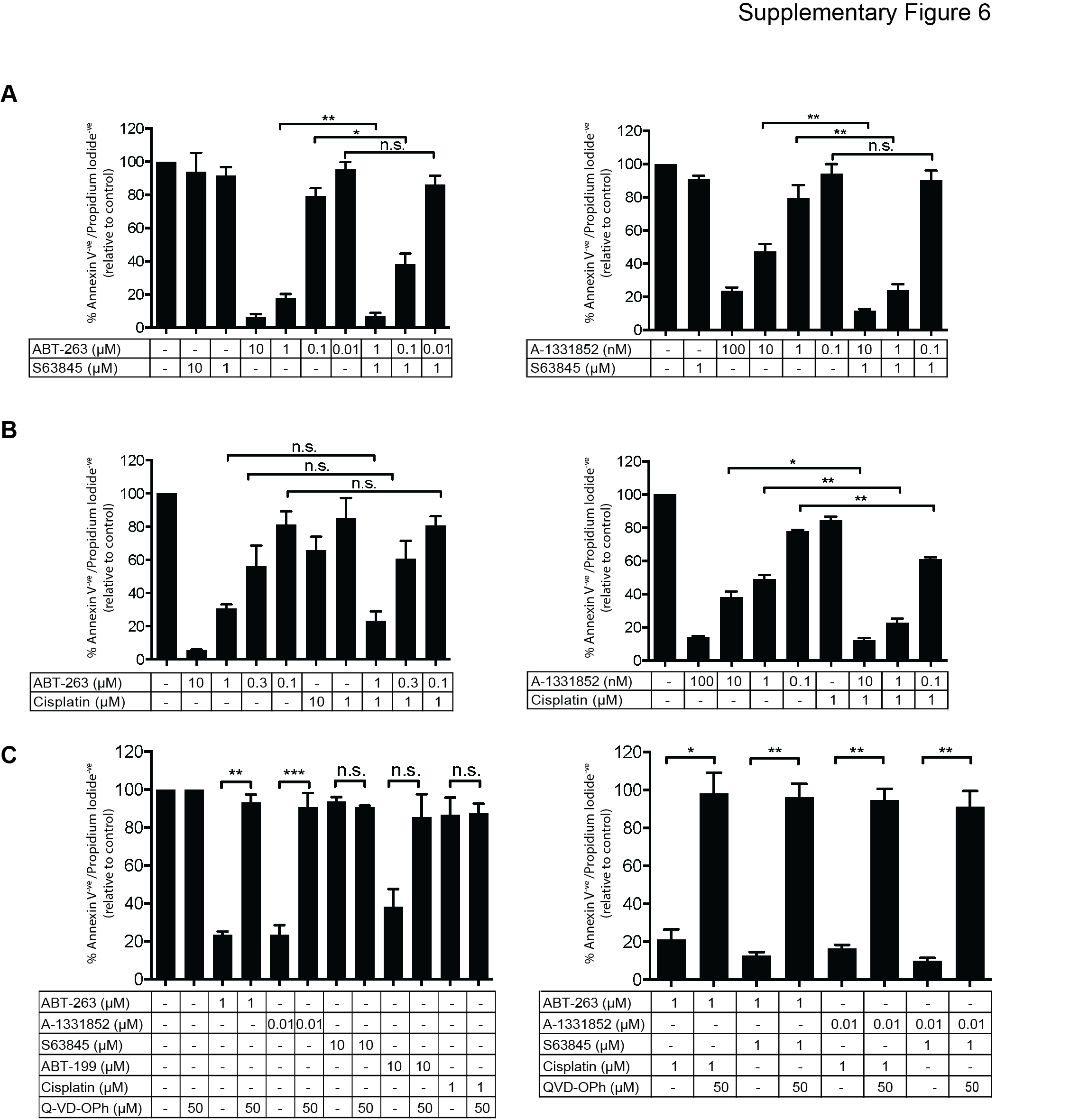

Supplement: Supplementary file 15 — Supplementary Figure 6 [file 41420_2020_348_MOESM15_ESM.png]

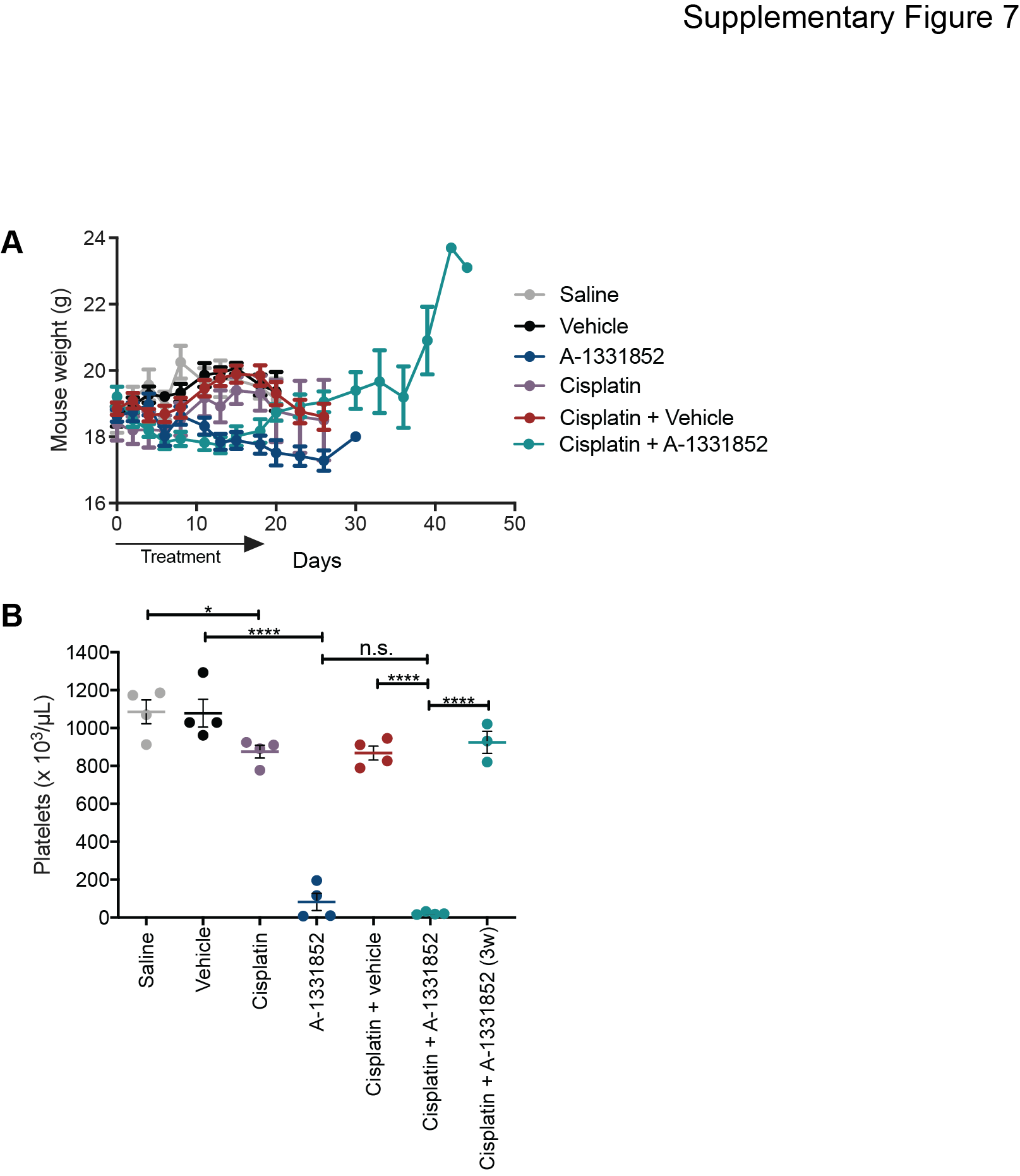

Supplement: Supplementary file 16 — Supplementary Figure 7 [file 41420_2020_348_MOESM16_ESM.png]

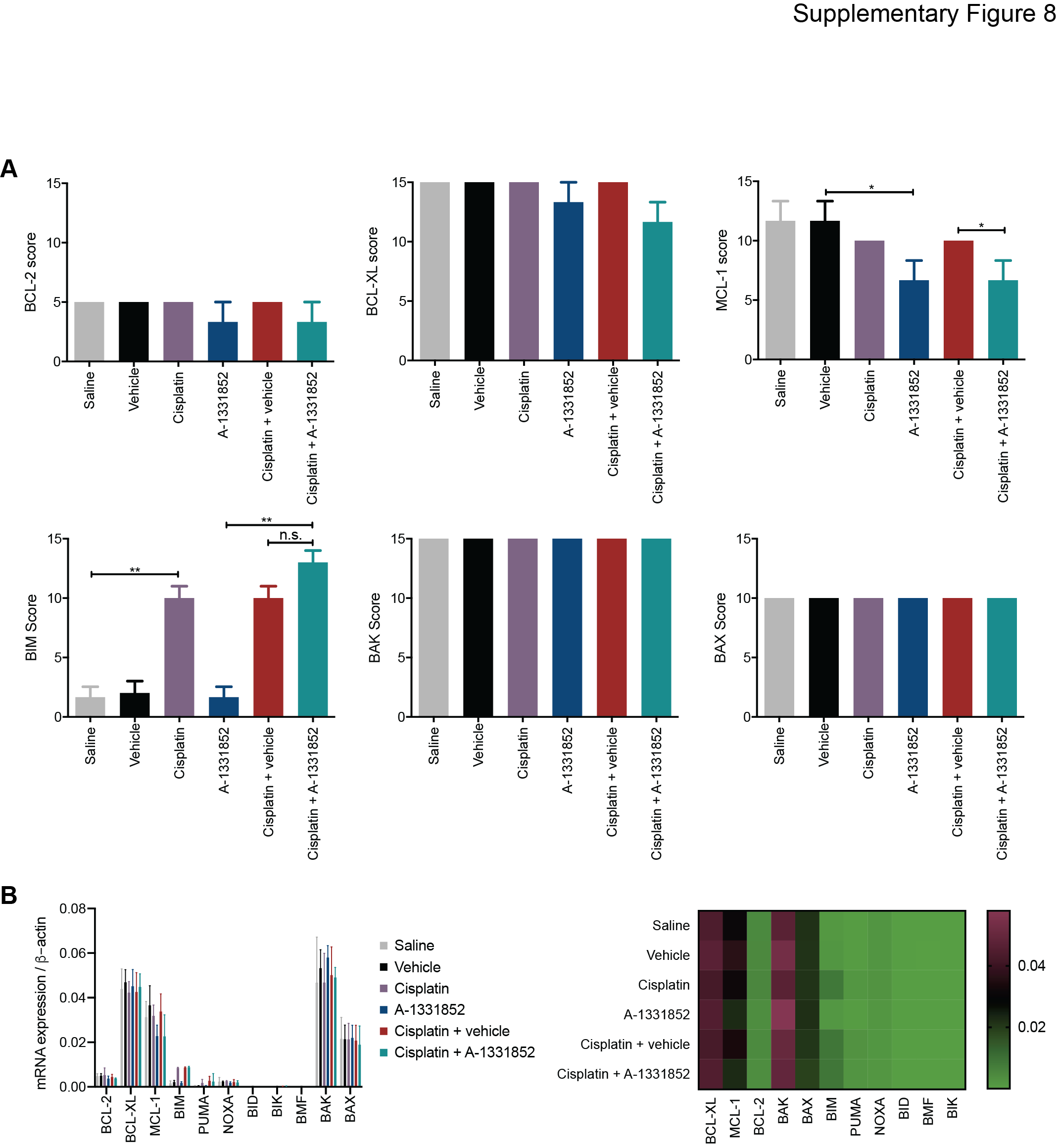

Supplement: Supplementary file 17 — Supplementary Figure 8 [file 41420_2020_348_MOESM17_ESM.png]

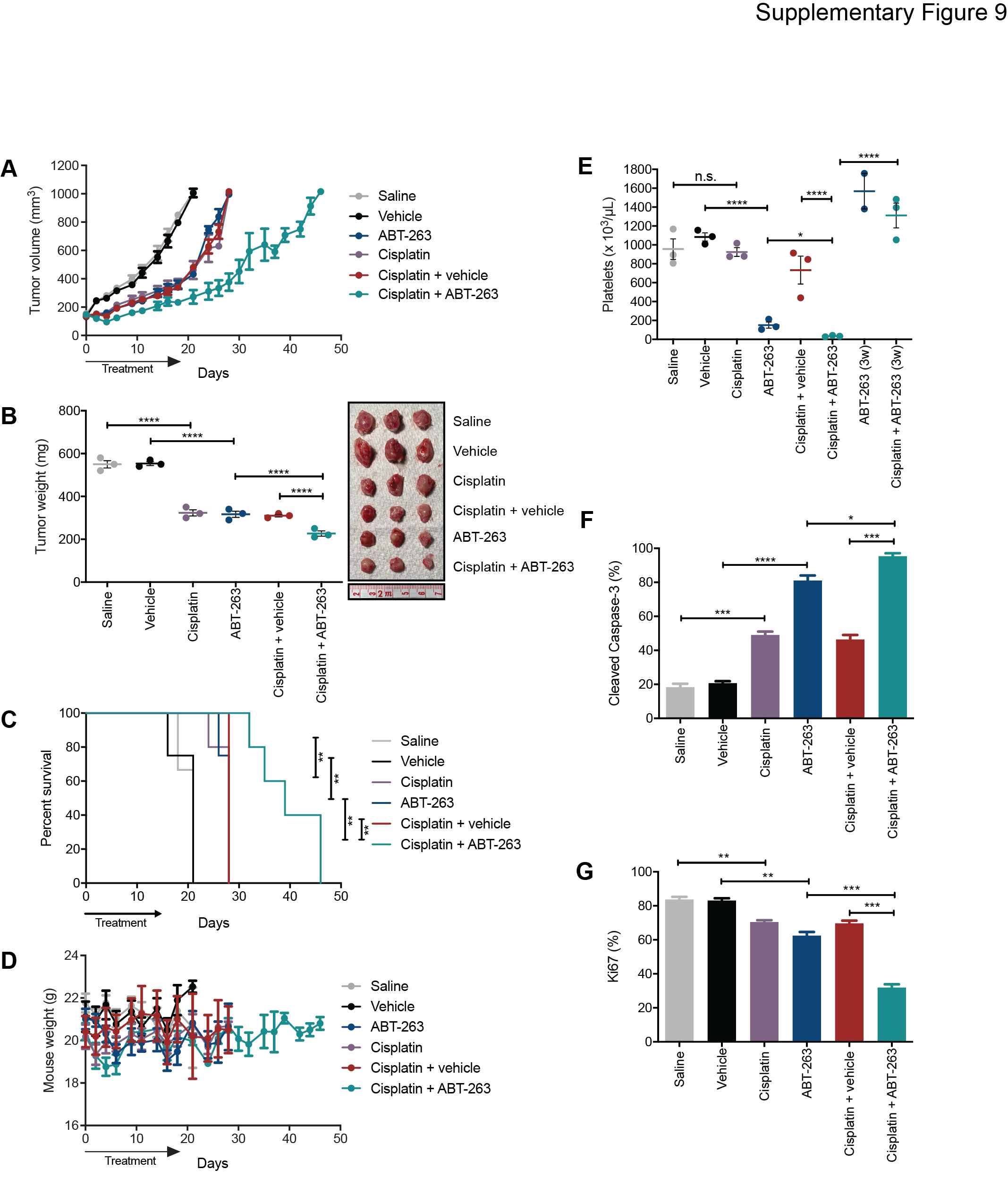

Supplement: Supplementary file 18 — Supplementary Figure 9 [file 41420_2020_348_MOESM18_ESM.png]

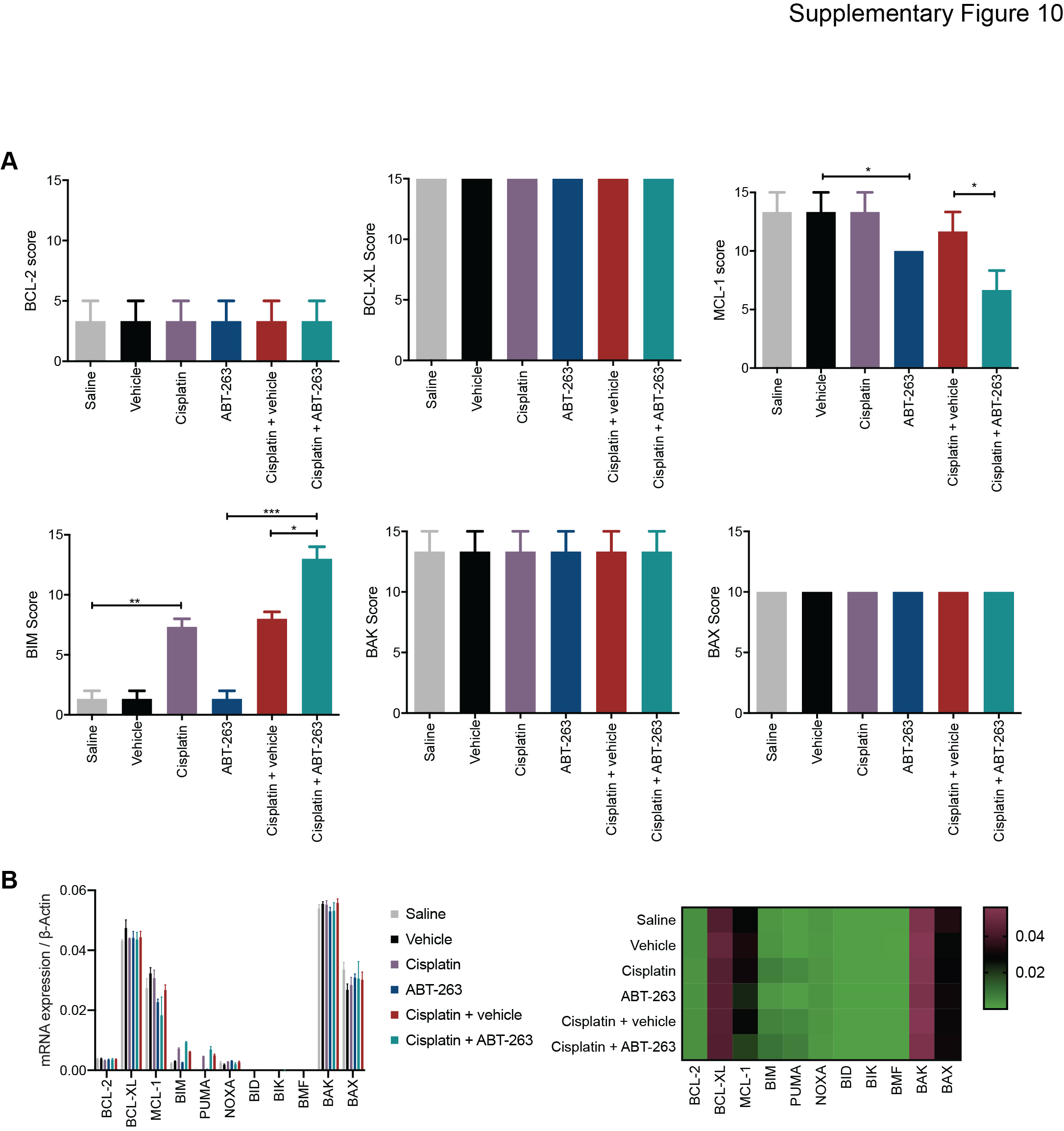

Supplement: Supplementary file 19 — Supplementary Figure 10 [file 41420_2020_348_MOESM19_ESM.png]

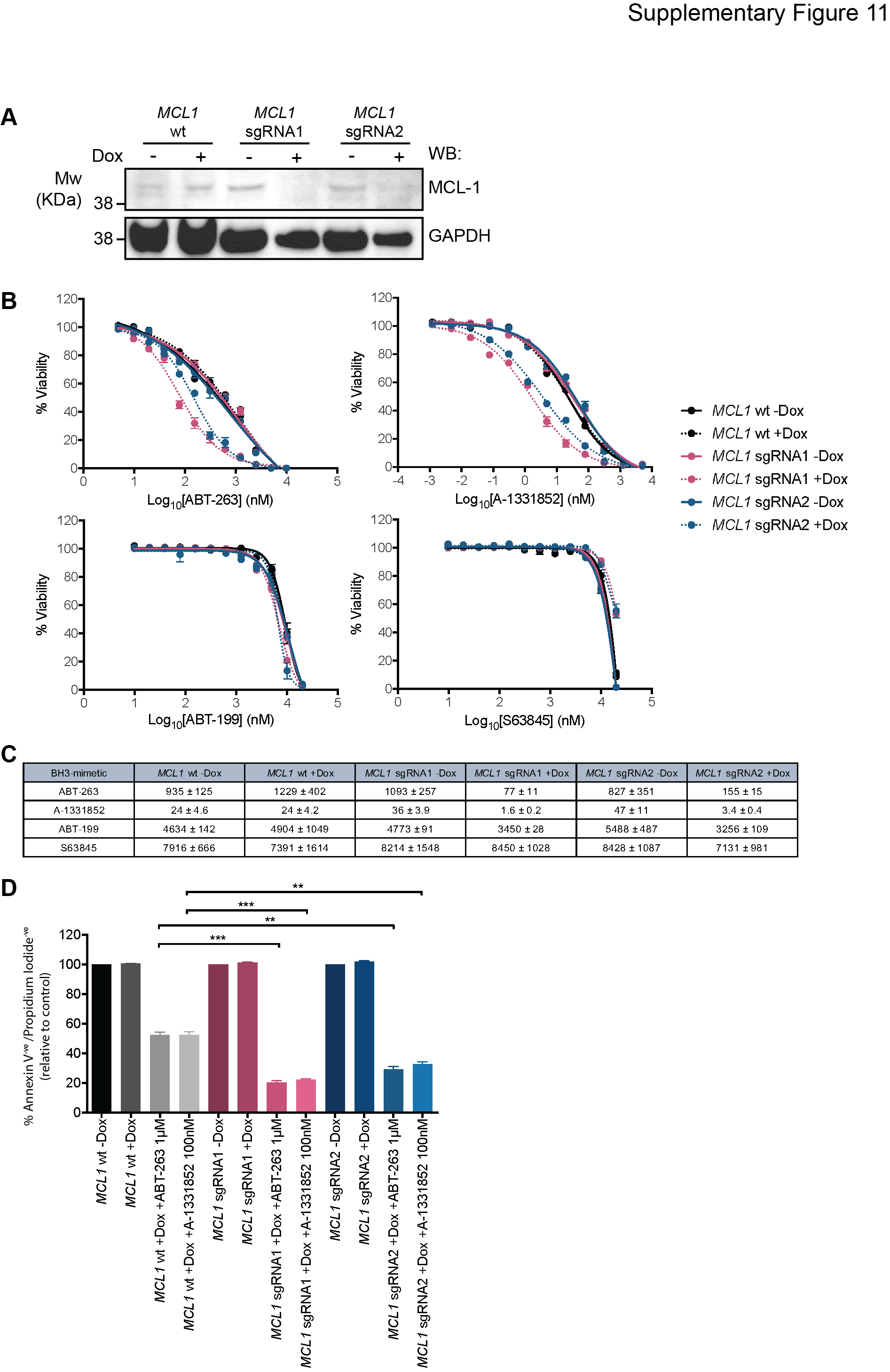

Supplement: Supplementary file 20 — Supplementary Figure 11 [file 41420_2020_348_MOESM20_ESM.png]

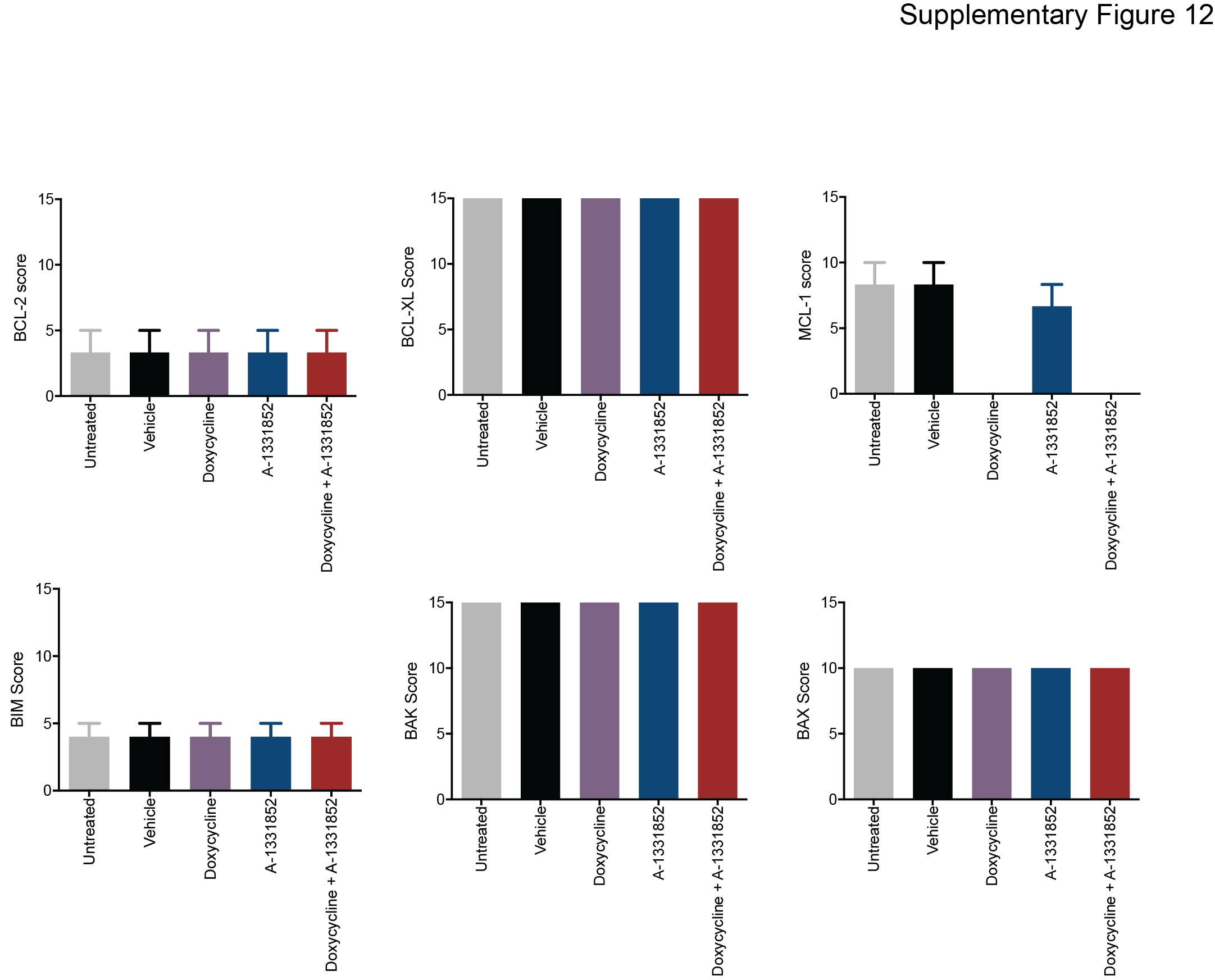

Supplement: Supplementary file 21 — Supplementary Figure 12 [file 41420_2020_348_MOESM21_ESM.png]

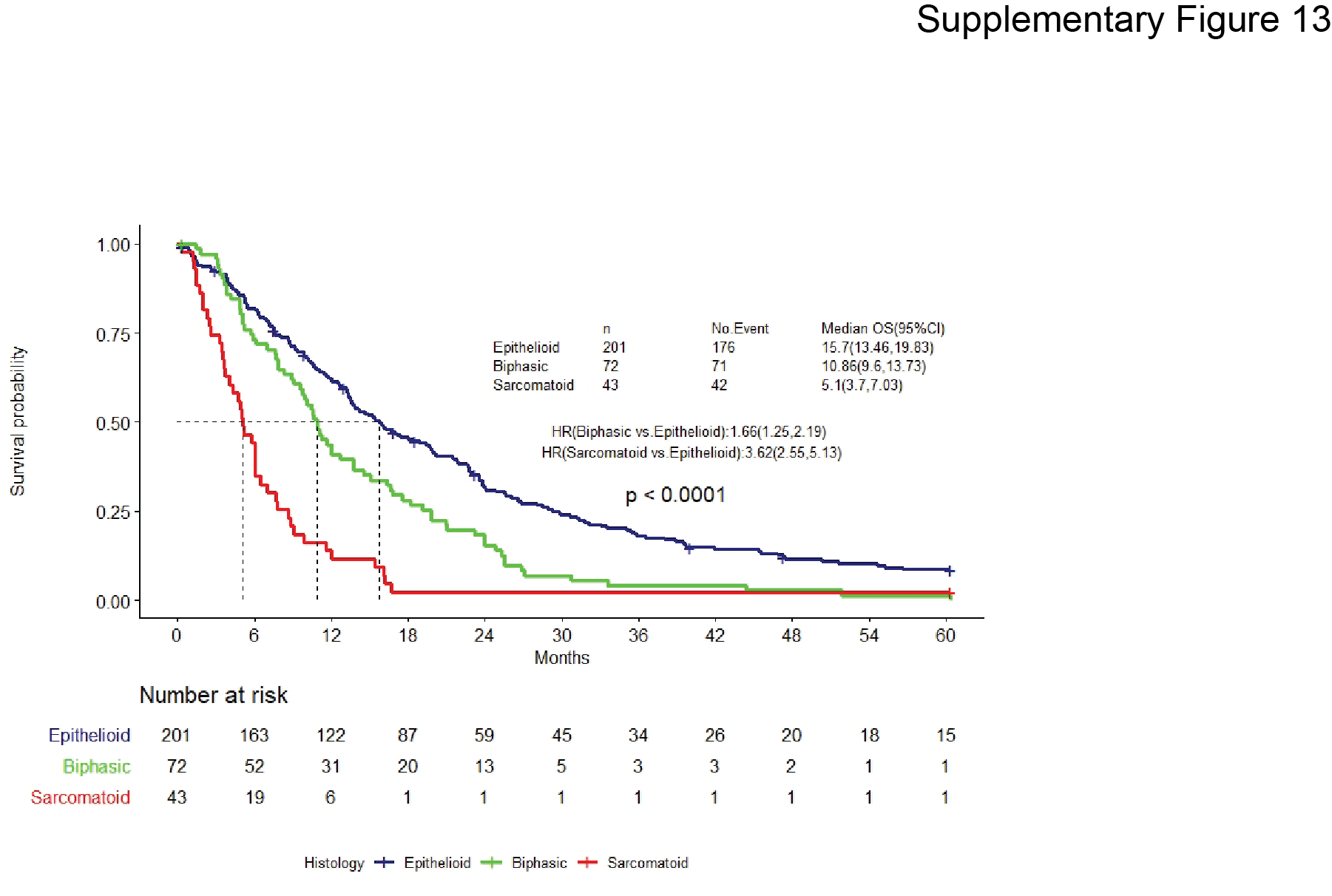

Supplement: Supplementary file 22 — Supplementary Figure 13 [file 41420_2020_348_MOESM22_ESM.png]

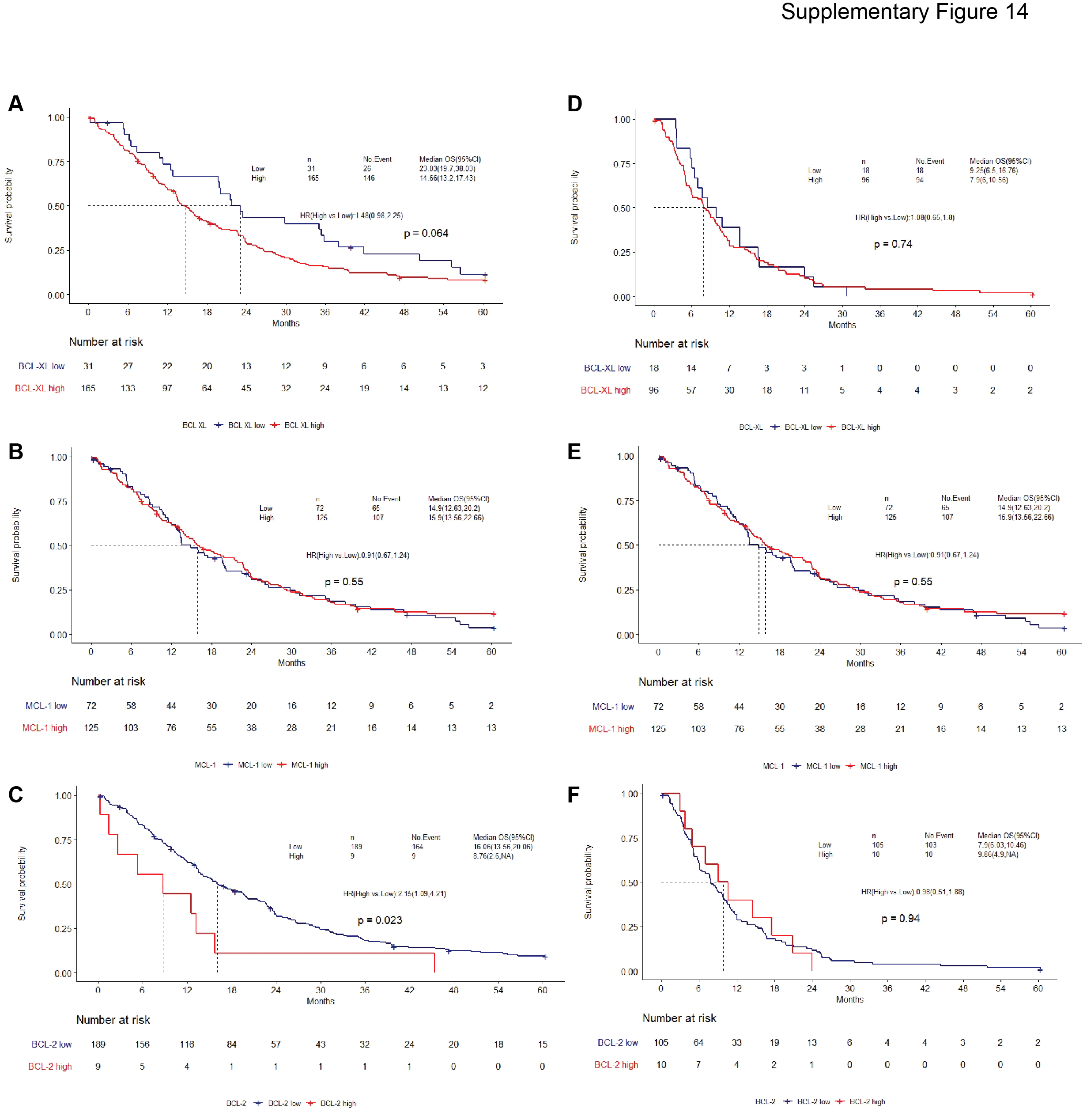

Supplement: Supplementary file 23 — Supplementary Figure 14 [file 41420_2020_348_MOESM23_ESM.png]
